# STING directly interacts with PAR to promote apoptosis upon acute ionizing radiation-mediated DNA damage

**DOI:** 10.1038/s41418-025-01457-z

**Published:** 2025-02-12

**Authors:** Yirong Sun, Saba R. Aliyari, Kislay Parvatiyar, Lulan Wang, Anjie Zhen, Wei Sun, Xiaobo Han, Adele Zhang, Ethan Kato, Helen Shi, Elena De Schutter, William H. McBride, Samuel W. French, Genhong Cheng

**Affiliations:** 1 CAS Key Laboratory of Regenerative Biology, Guangdong Provincial Key Laboratory of Stem Cell and Regenerative Medicine, Guangzhou Institutes of Biomedicine and Health, Chinese Academy of Sciences, Guangzhou, China; 2 Department of Microbiology, Immunology and Molecular Genetics, University of California–Los Angeles, Los Angeles, CA USA; 3 Department of Microbiology & Immunology, Tulane University School of Medicine, New Orleans, LA USA; 4 Department of Radiation Oncology, University of California–Los Angeles, Los Angeles, CA USA; 5 Department of Pathology and Laboratory Medicine, David Geffen School of Medicine, University of California–Los Angeles, Los Angeles, CA USA

**Keywords:** Gastrointestinal diseases, Cell death and immune response

## Abstract

Acute ionizing radiation (IR) causes severe DNA damage, leading to cell cycle arrest, cell death, and activation of the innate immune system. The role and signaling pathway of stimulator of interferon genes (STING) in IR-induced tissue damage and cell death are not well understood. This study revealed that STING is crucial for promoting apoptosis in response to DNA damage caused by acute IR both in vitro and in vivo. STING binds to poly (ADP‒ribose) (PAR) produced by activated poly (ADP‒ribose) polymerase-1 (PARP1) upon IR. Compared with that in WT cells, apoptosis was suppressed in *Sting*^*gt-/gt-*^ cells. Excessive PAR production by PARP1 due to DNA damage enhances STING phosphorylation, and inhibiting PARP1 reduces cell apoptosis after IR. In vivo, IR-induced crypt cell death was significantly lower in *Sting*^*gt-/gt-*^ mice or with low-dose PARP1 inhibitor, PJ34, resulting in substantial resistance to abdominal irradiation. STING deficiency or inhibition of PARP1 function can reduce the expression of the proapoptotic gene PUMA, decrease the localization of Bax on the mitochondrial membrane, and thus reduce cell apoptosis. Our findings highlight crucial roles for STING and PAR in the IR-mediated induction of apoptosis, which may have therapeutic implications for controlling radiation-induced apoptosis or acute radiation symptoms.

**Figure:**
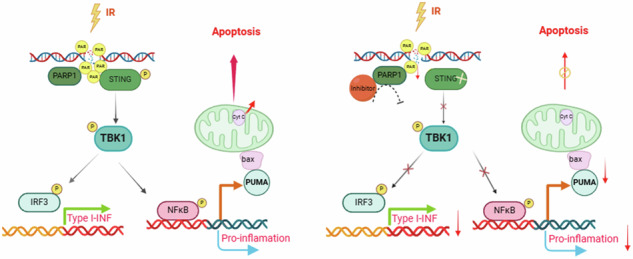
STING responds to acute ionizing radiation-mediated DNA damage by directly binding to poly (ADP-ribose) (PAR) produced by activated poly (ADP-ribose) polymerase-1 (PARP1), and mainly induces cell apoptosis through Puma-Bax interaction. STING deficiency or reduced production of PAR protected mice against Acute Radiation Syndrome.

STING responds to acute ionizing radiation-mediated DNA damage by directly binding to poly (ADP-ribose) (PAR) produced by activated poly (ADP-ribose) polymerase-1 (PARP1), and mainly induces cell apoptosis through Puma-Bax interaction. STING deficiency or reduced production of PAR protected mice against Acute Radiation Syndrome.

## Introduction

High-dose radiotherapy for pelvic and abdominal tumors can lead to gastrointestinal syndrome (GIS) [1]. Nuclear accidents or bioterrorism may potentially result in the exposure of thousands of individuals to high doses of ionizing radiation (IR), ultimately causing acute radiation syndrome (ARS), which is characterized by substantial damage to internal organs and tissues that contain highly proliferating cells, including those in the bone marrow and gastrointestinal (GI) tract [2–4]. Exposure to acute IR elicits severe DNA damage to highly proliferating cells and triggers the activation of a variety of cellular responses, including the DNA repair process and/or cell death programs [5, 6]. Indeed, the apoptosis of endothelial cells in the intestinal vasculature [5, 7] has been demonstrated to play an important role in the GIS. On the other hand, IR-mediated cell death and its associated immune responses are also important for radiation therapy against diseases such as cancers [3, 6].

Despite decades of research, the key molecular mechanisms underlying cell death in radiation-induced GIS remain controversial, and there is a lack of effective drugs and methods for treatment. Recently, research has revealed that the unconventional prefoldin RPB5 interactor (URI) and p53 overexpression protect against GIS [2, 8]. Kirsch et al. reported that GI epithelial cell death is regulated by p53 but independent of apoptosis, indicating that p53-mediated cell apoptosis is not sufficient to cause GIS [2]. The complex mechanisms of GIS remain poorly understood. Therefore, it is necessary to identify the targets required to reduce intestinal damage during IR and determine their function.

Studies have revealed that acute IR causes the leakage of damaged DNA species from the nuclear or mitochondrial compartments [9, 10]. Poly (ADP‒ribose) polymerase (PARP) functions as the first responder in both chromosomal and mitochondrial DNA damage. It detects single- and double-stranded DNA breaks (DSBs) and catalyzes the cleavage of NAD^+^ into nicotinamide and ADP-ribose [11, 12]. PARP-1 has dual regulatory functions in cell death and survival, but how PARP-1 signaling regulates and switches between DNA repair and cell death is unclear. The addition of poly (ADP‒ribose) (PAR) chains regulates the recruitment of multiple factors to DNA damage sites, triggering DNA repair or programmed cell death [13, 14]. DNA DSBs can be repaired by homologous recombination or by the nonhomologous end-joining (NHEJ) pathway at different stages of cell cycle [15]. DNA-PK mutant mice are sensitive to GI-ARS because they are unable to repair DSBs, and DNA-PK and p53 compound mutant mice are more sensitive to IR [16], suggesting that a combined approach to regulate these two pathways may be beneficial in the treatment of GI-ARS. Cytosolic detection of these aberrant DNA species by pattern recognition receptors can activate signaling events to produce type I interferon (IFN-I) and proinflammatory cytokines [17–19]. Notably, the induction of the host IFN-I response upon DNA damage requires stimulator of interferon genes (STING) [20–22]. However, the role of STING in regulating IR-induced tissue damage and cell death remains poorly defined [23–25].

Here, we identified an essential role for STING in facilitating IR-mediated tissue damage and apoptosis in an IFN-I-independent manner. Furthermore, we present evidence that the association of STING with poly (ADP-ribose) is required to elicit IR-induced programmed cell death. Our study suggests that the inhibition of STING activity or partial inhibition of PARP1 is a potentially important target for the treatment of radiation injury. Conversely, the activation of PAR-STING by radiotherapy or agonists can also provide new perspectives for tumor treatment [26, 27].

## Results

### STING deficiency protected mice against acute radiation syndrome and increased cell survival after ionizing radiation

**Fig. 1:**
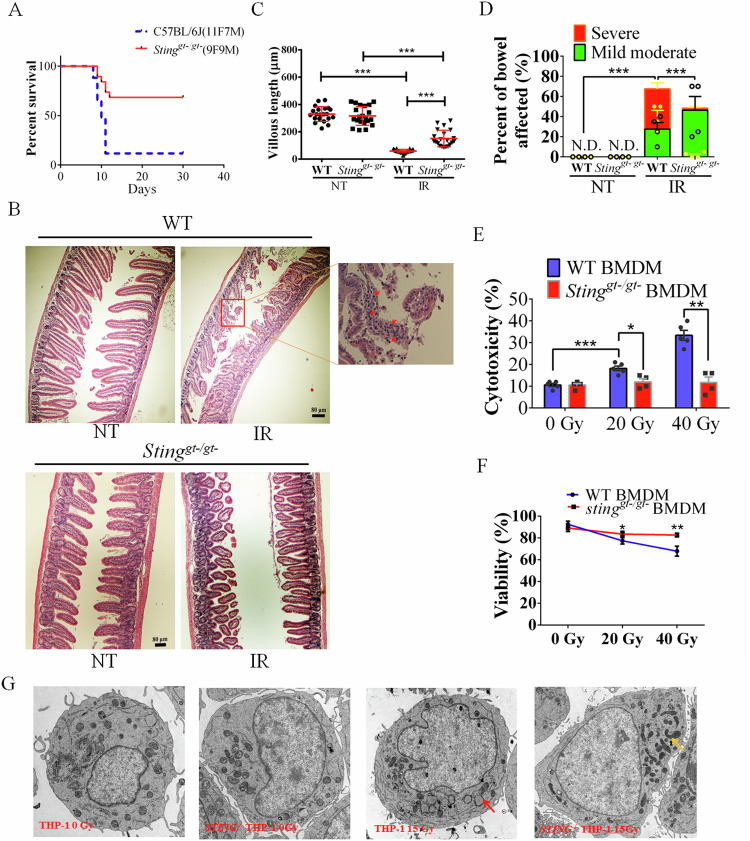
STING deficiency improves survival and protects against IR-mediated tissue damage. **A** Survival of WT C57BL/6 J and *Sting*^*gt-/gt-*^ C57BL/6 J after exposure to 16.7 Gy SBI. F, female; M, male. **B** H&E staining of WT C57BL/6 J and *Sting*^*gt-/gt-*^ C57BL/6 J intestines with (IR) and without (NT) abdominal radiation at 4 dpi (4 days post irradiation). **C** Villus height of the intestine in WT C57BL/6 J and *Sting*^*gt-/gt-*^ C57BL/6 J mice at 4 dpi with (IR) and without (NT) abdominal radiation. The data are presented as the means ± SEMs (*n* = 5; two-way ANOVA; *p < 0.05; **p < 0.01; ***p < 0.001). **D** Crypt loss villous atrophy (percent of bowel affected) after IR in WT and *Sting*^*gt-/gt-*^ mice with and without abdominal radiation. N. D., Not detected. ***p < 0.001. **E** The level of cytotoxicity of BMDMs at 6 h postirradiation (6 hpi) was measured by LDH release into the supernatant. **F** Survival rates of WT and *Sting*^*gt-/gt-*^ derived BMDMs exposed to 20 and 40 Gy radiation at 6 hpi. The data are reported as the means ± standard deviations (s.d.) (*n* = 6; *p < 0.05; **p < 0.01; ***p < 0.001). **G** THP-1 and *STING*^*-/-*^ THP-1 cell morphology was detected via transmission electron microscopy (TEM) (Hitachi HT-7800).

The GI tract and bone marrow contain cells with a high turnover rate to replenish dying cells and establish a balance between cell death and proliferation. Therefore, a higher rate of proliferation renders these cells more susceptible to severe DNA damage after exposure to IR [6, 10]. To investigate the role of STING in IR-mediated DNA damage and the molecular mechanisms underlying IR-induced cell death, we used a murine model of abdominal/subtotal irradiation (SBI). First, we evaluated the survival of *Sting*^*gt-/gt-*^ mice (mice harboring the null I199N STING mutation on the C57BL/6 J background) and WT mice (C57BL/6 J) after abdominal irradiation. These mice were exposed to a high dose of IR targeting the abdomen (SBI) to sequester hematopoietic cells from the GIS [2, 4]. As expected, most of the WT animals succumbed to abdominal SBI. Surprisingly, the survival rate of *Sting*^*gt-/gt-*^ mice was significantly greater after abdominal SBI treatment. A total of 89% of the WT mice died (16 of 18 mice died) within 10 days after exposure to IR, whereas 67% (12 of 18 mice survived) of the *Sting*^*gt-/gt-*^ mice survived IR for 30 days (Fig. 1A).

Next, we evaluated SBI-mediated damage to the colon and intestines in irradiated animals (Figure. S1A, Fig. 1B). Histological analysis revealed greater abdominal damage in WT mice than in *Sting*^*gt-/gt-*^ mice (Fig. 1B). Accordingly, the average intestinal villous height of *Sting*^*gt-/gt-*^ mice was significantly greater than that of WT animals post-IR (Fig. 1C), and the average villous height of *Sting*^*gt-/gt-*^ mice was 152 ± 62 μm, whereas that of WT mice was 65 ± 9 μm on day 4 after IR. Crypt loss was 23% with severe villous atrophy in WT mice, whereas little villous atrophy was detected, and only 46% crypt loss with middle to moderate villous atrophy was observed in *Sting*^*gt-/gt-*^ mice after SBI (Fig. 1D). Increased inflammation was observed in WT mice after irradiation, whereas no obvious inflammation was observed in STING-inactivated mice, which may be due to the reduced production of proinflammatory factors caused by STING inactivation (Fig. 1B).

We further evaluated the role of STING in the survival of bone marrow-derived macrophages (BMDMs) after exposure to IR. We found that *Sting*^*gt-/gt-*^-derived BMDMs presented lower cytotoxicity and greater survival than WT control BMDMs did (Fig. 1E, F). In addition to the role of STING in primary BMDMs, we examined the role of STING in radiation-induced cytotoxicity in immortalized murine macrophage lines (J2 BMDMs). WT J2 BMDMs displayed a pronounced loss of nuclei with increasing IR doses, which was largely absent in *Sting*^*gt-/gt-*^ J2 BMDMs (Figure. S1B, C). Similar results were obtained when mouse embryonic fibroblasts (MEFs) were exposed to IR (Figure. S1D), where the survival rate of *Sting*^*-/-*^ MEFs was greater than that of WT MEFs. We further investigated the viability of WT and *STING*^-/-^ human THP-1 monocytes after exposure to IR. Our results revealed that the loss of STING significantly increased cell survival (Figure. S1E, F) after irradiation.

The transmission electron microscopy (TEM) results of THP-1 cell morphology also revealed apoptosis and a decrease in the number of mitochondria in THP-1 cells after irradiation, but the mitochondria of STING knockout cells remained relatively intact and even elongated (Fig. 1G). Acute endoplasmic reticulum stress has been reported to induce protective mitochondrial morphological elongation [28, 29].

Our data revealed that STING deficiency significantly increased cell survival after IR and mouse survival after SBI (Fig. 1 and Figure. S1). These results confirmed that STING dysfunction can promote mouse and cell survival after IR.

### Loss of STING function suppresses IR-mediated apoptosis both in vitro and in vivo

**Fig. 2:**
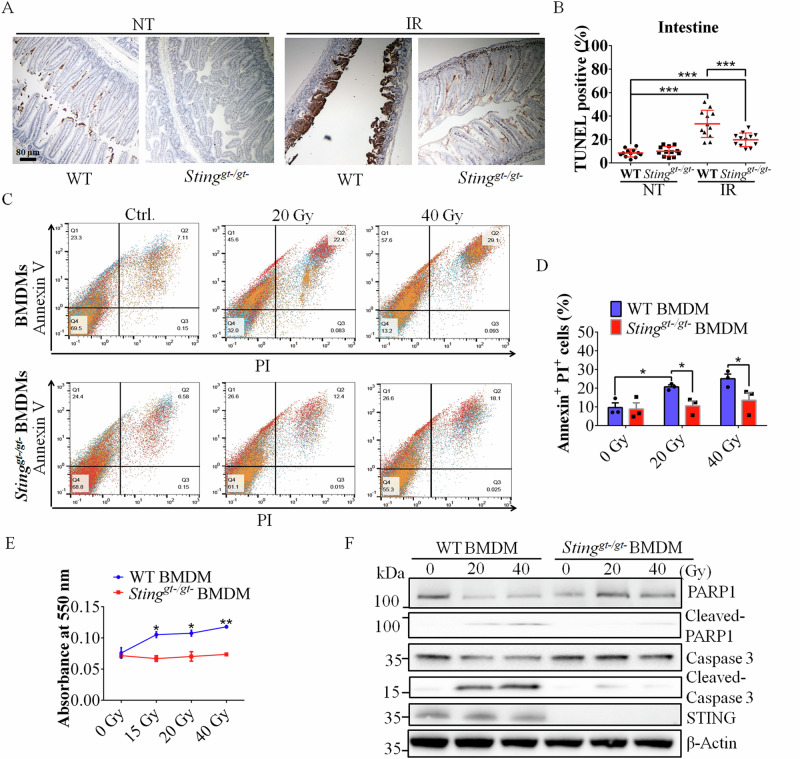
Loss of STING function suppresses the apoptotic pathway. **A** TUNEL staining of the intestines of WT and *Sting*^*gt-/gt-*^ mice at 0 and 4 dpi. **B** Quantification of TUNEL-positive cells in the intestine after SBI at 0 and 4 dpi. The percentages of TUNEL-stained areas in all intestine regions were statistically analyzed. **C** BMDM viability was measured by annexin V-FITC-propidium iodide staining of WT and *Sting*^*gt-/gt-*^ BMDMs after exposure to the indicated dose of IR. **D** Quantification of Annexin V and propidium iodide labeling. **E** The number of apoptotic cells after exposure to the indicated dose of IR. Apoptotic cells were quantified with a Cell-APOPercentage™ apoptosis kit at 550 nm. **F** Evaluation of PARP1 and caspase 3 cleavage in WT BMDMs and *Sting*^*gt-/gt-*^ BMDMs after exposure to the indicated dose of IR by Western blotting. The data are presented as the means ± SEMs (two-way ANOVA; *p < 0.05; **p < 0.01; ***p < 0.001).

To determine whether programmed cell death mediates abdominal injury in mice after exposure to SBI, we examined the affected tissues via terminal deoxynucleotidyl transferase dUTP nick end labeling (TUNEL) staining. The results of the TUNEL assay indicated the occurrence of SBI-mediated apoptosis in the colon and intestine of both WT and *Sting*^*gt-/gt-*^ mice exposed to SBI (Fig. 2A, B and Figure. S2A, B). While the level of apoptosis in colon cells was not significantly different between WT and *Sting*^*gt-/gt-*^ mice (Figure. S2A, B), the level of apoptosis was significantly greater in WT intestinal cells than in *Sting*^*gt-/gt-*^ mice (Fig. 2A, B). Our results also confirmed that the intestine is more sensitive to radiation than the colon is.

Next, we investigated whether STING deficiency effects ROS after irradiation. Notably, there was no significant difference in the levels of reactive oxygen species (ROS) in wild-type and *STING*^-/-^ THP-1 cells after exposure to IR (Figure. S2C, D), indicating that STING deletion does not affect the amount of ROS produced after IR.

We further investigated the rate of IR-mediated apoptosis in BMDMs derived from WT or *Sting*^*gt-/gt-*^ mice via flow cytometry and biochemical analysis. Consistent with our results above, the level of apoptosis was lower in *Sting*^*gt-/gt-*^ BMDMs than in WT BMDMs (Fig. 2C–E). Activated caspase-3 cleaves proteins, including the DNA repair protein PARP-1, thus promoting apoptosis [11, 30]. Interestingly, the rates of both caspase 3 activation and PARP1 cleavage were increased after IR in a dose-dependent manner in WT BMDMs but not in *Sting*^*gt-/gt-*^ BMDMs (Fig. 2F, Figure. S2E). Similar results were also obtained when MEFs and THP-1 cells were exposed to IR. *Sting*-deficient cells were significantly resistant to IR-mediated cell death (Figure. S3A, B).

### STING interacts with PAR after IR

**Fig. 3:**
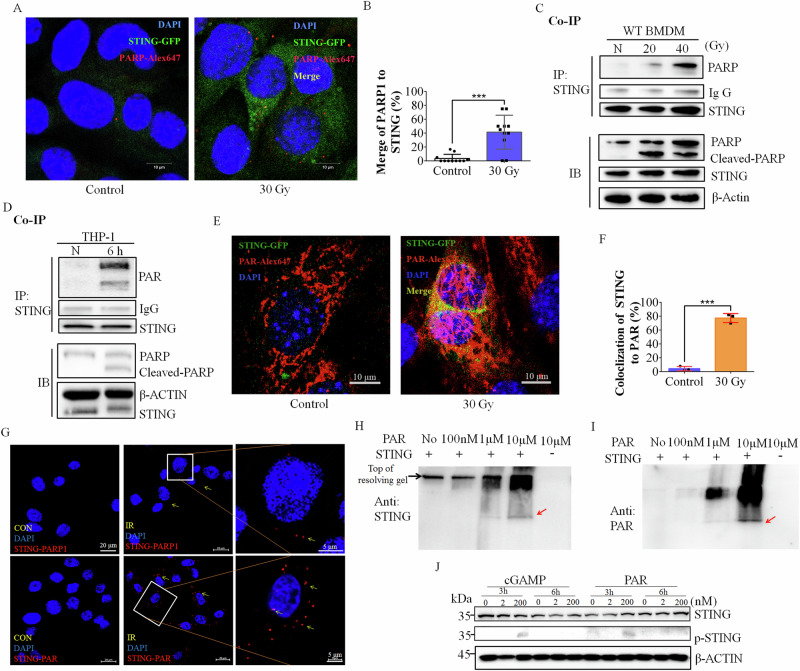
The STING response to DNA damage after IR is associated with PAR-PARP1. **A** Confocal images of the interactions between PARP1-Alex647 and STING-GFP via a Zeiss Elyra-7 microscope. **B** Quantification of the interaction between PARP1-Alex647 and STING-GFP via ImageJ software. Colocalization analysis was carried out via Manders’ colocalization coefficient (MCC) method. **C** Evaluating the association of STING with PARP1 in BMDMs. The cell lysates were immunoprecipitated with anti-STING beads, followed by immunoblotting with the indicated antibodies. **D** Coimmunoprecipitation and immunoblotting of the interaction of STING with PAR in THP-1 cells subjected to 30 Gy IR for 6 h. The cell lysates were immunoprecipitated with anti-STING beads, followed by immunoblotting with the indicated antibodies. **E**, **F** Microscopy images of PAR-Alex647 and STING-GFP interactions captured with ZEISS Elyra-7 (**E**) and quantified with ImageJ software (**F**). **G** Validation of the ability of PLA to detect the proximity between STING and PAR or PARP1 in MDA-MB-231 cells. Nuclei were stained with DAPI (blue); PLA was performed for STING and PAR or PARP1 (red). CON, non-irradiated. **H**, **I** The binding of STING and PAR in vitro was analyzed by nondenaturing polyacrylamide gel electrophoresis and immunoblotting with anti-STING (**H**) and anti-PAR (**I**) antibodies. **J** THP-1 cells were transfected with the indicated concentrations of PAR and cGAMP via Lipofectamine. PAR (0.2 μM) induced the phosphorylation of STING. The data are presented as the means ± SEMs (unpaired Student’s t test; ***, *p* < 0.001).

PARP1 functions as a first responder in the DNA damage response, which detects single- and double-stranded DNA breaks [31]. We used BMDMs, THP-1 cells, and *Sting*^*-/-*^ MEFs reconstituted with STING-GFP to address the relationship between STING and PARP1. We found that the expression of PARP1 increased in BMDMs and THP-1 cells after IR. Interestingly, after IR, PARP1 and STING colocalized (Fig. 3A, B), whereas cGAS did not colocalize with STING (Figure. S3C). In line with our imaging analysis, coimmunoprecipitation assays revealed that IR triggered the interaction of STING with PARP1 in BMDMs and THP-1 cells (Fig. 3C, Figure. S3D). Coimmunoprecipitation assays in *STING*^-/-^ THP-1 cells reconstituted with MYC-tagged STING further confirmed that PARP1 and STING interacted upon IR (Figure. S3E). These results indicate that the STING response to IR-mediated DNA damage is associated with PARP1.

As PAR is produced by PARP1 [32], we investigated the possible interaction of PAR with STING. Indeed, we found that PAR immunoprecipitated with STING (Fig. 3D). Immunofluorescence analysis also revealed that PAR colocalized with STING after IR (Fig. 3E, F). We further validated the colocalization of STING with PAR via a proximity ligation assay (PLA), a highly specific and sensitive immunohistochemical tool for identifying the physical closeness of proteins [33]. PLA images revealed that most cells exhibited easily detectable red fluorescence, indicating that PAR and STING were in close proximity after IR (Figure. S3F), while anti-PARP1 and anti-STING together also presented some smaller red fluorescent spots, but these spots were smaller and fewer in number than those observed when the PLA assay were performed in the presence of both anti-PAR and anti-STING (Fig. 3G and Figure. S3F). However, no red fluorescent dots were detected in the cells without radiation. The results of the PLA further suggest that STING is associated with PAR after IR. Some PARP1 proteins are still associated with PAR because of the synthesis of PAR. We deduced that some PARP1 and STING proteins are very close because they co-interact with PAR.

Furthermore, we mixed purified human STING with different concentrations of PAR in vitro. The products were analyzed by nondenaturing PAGE and immunoblotting analysis with anti-STING and anti-PAR antibodies. The results indicated that STING and PAR presented overlapping binding, and PAR binding to STING altered PAR-STING electrophoretic migration due to the negative charge of PAR [34] (Fig. 3H, I). Therefore, these results further confirmed that in response to IR-mediated DNA damage, STING is associated with the PARP1 and PAR chains.

**Fig. 4:**
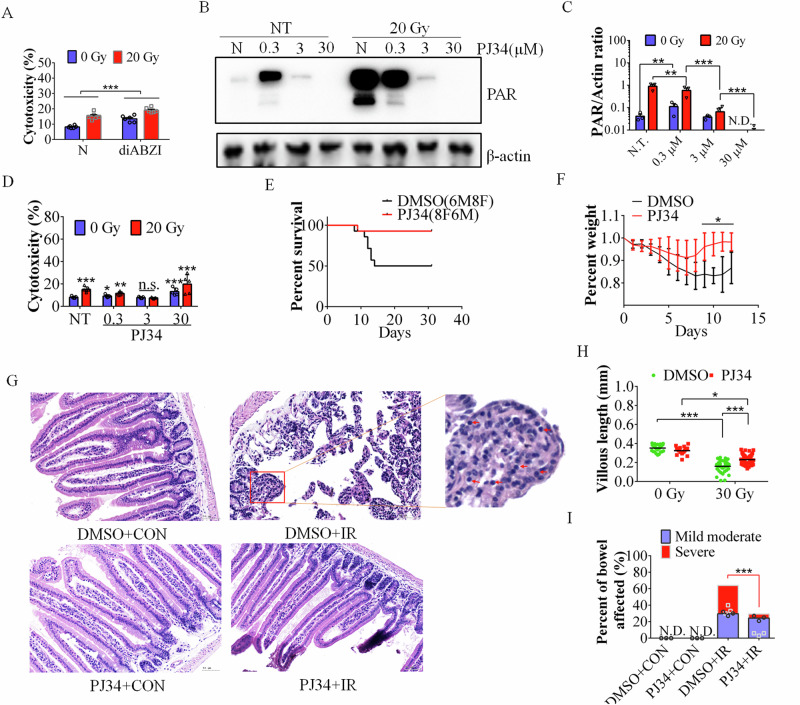
The PARP1 inhibitor PJ34 protects cells and mice against IR. **A** BMDMs treated with the control vehicle or diABZI (1 μg/ml) were subjected to 20 Gy radiation. The level of cytotoxicity of BMDMs at 6 hpi was measured by LDH release into the supernatant. **B**, **C** Evaluation of PAR in cells treated with vehicle or the indicated dose of PJ34 by immunoblotting (**B**), and the relative concentrations were calculated (**C**). N.D., not detected. **D** Evaluation of BMDM viability after treatment with the indicated dose of PJ34 (μM) with or without IR (20 Gy). **E**, **F** Compared with the control (DMSO), 1 mg/kg PJ34 increased the resistance of the mice to 16.2 Gy SBI (**E**) and (**F**) inhibited weight loss after 8 days. Male mice with the same age were randomly divided into two groups. **G** H&E staining of C57BL/6 J mouse intestines subjected to IR or not subjected to IR (CON) and treated with 1 mg/kg PJ34 or the control vehicle (NT) at 4 dpi. **H** Villus height of intestines subjected to the indicated dose of SBI and treated with PJ34 or the vehicle control. **I** Crypt loss villous atrophy (percent of bowel affected) after IR in mice with and without abdominal radiation and PJ34 (1 mg/kg) treatment. The data are presented as the means ± SEMs (*n* = 5; two-way ANOVA; *, *p* < 0.05; **, *p* < 0.01; ***, *p* < 0.001).

STING phosphorylation plays a key role in innate immune activation and the cell apoptosis signaling pathway [22, 24, 35]. To study the role of IR-mediated activation of the STING signaling pathway, we investigated the pattern of STING phosphorylation after IR. The exposure of WT BMDMs to increasing levels of IR was correlated with increased STING phosphorylation (Figure. S3G). To further investigate the effect of PAR on STING signaling, cells were treated with PAR. Western blot analysis revealed that STING phosphorylation was significantly increased after 3 h of 200 nM PAR treatment and cGAMP treatment, but there was no obvious phosphorylation after 6 h, which may be due to PAR instability (Fig. 3J). Next, we investigated PARP1 cleavage in the presence of the STING agonist diABZI, which is reported to activate STING and induce apoptosis [36]. Indeed, diABZI induced the phosphorylation of STING, increased PARP1 cleavage (Figure. S3H), and increased cell cytotoxicity (Fig. 4A), suggesting that STING phosphorylation is associated with an increased rate of apoptosis, which is consistent with our previous report [26].

### Partial inhibition of PARP1 by PJ34 protects cells and mice against IR

The negatively charged polymer of PAR is synthesized by PAR polymerases (PARPs) from nicotinamide adenine dinucleotide (NAD^+^), which regulates not only cell survival but also cell death programs [37, 38]. Therefore, we studied the effects of different concentrations of PJ34 on PAR expression and cell survival after IR. The expression of PAR significantly increased upon IR and decreased with increasing concentrations of PJ34 (Fig. 4B, C). Cell cytotoxicity analysis revealed that 3 μM PJ34 protected cells from IR-induced death (Fig. 4D).

Based on the in vitro experimental results, we used the PARP inhibitor PJ34 to treat irradiated C57BL/6 J WT mice two hours after radiation (Figure. S4A). PJ34 significantly improved the survival rate of the mice after radiation (Fig. 4E). After exposure to IR, 93% (13 mice survived in 14 mice) of the PJ34-treated mice survived, whereas 50% of the control mice died within two weeks (7 mice died in 14 mice). Body weight loss significantly decreased in the mice without PJ34 treatment (Fig. 4F). After 20 days, most of the mice in the PJ34 treatment group started to move normally, but those in the control group still curled together (Figure. S4B). Fewer mice were observed to have hair loss in the PJ34-treated group than in the untreated group (Figure. S4B, C).

H&E staining revealed less abdominal damage in the mice with PJ34 than in the control mice (Fig. 4G–I). The intestinal villous height of the PJ34-treated mice was greater than that of the untreated mice (Fig. 4G, H). Crypt loss with more severe villous atrophy in the intestines was observed in untreated animals, whereas less severe villous atrophy was detected, and less crypt loss with middle to moderate villous atrophy was observed in PJ34-treated mice after SBI (Fig. 4G, I).

**Fig. 5:**
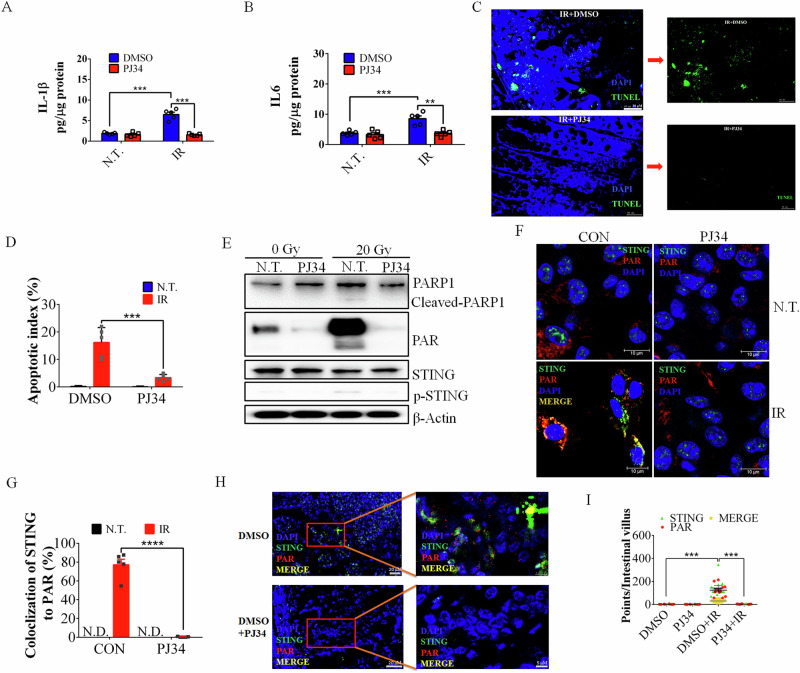
A precise dosage of PJ34 could reduce small intestinal tissue damage and apoptosis. **A**, **B** PJ34 blunts the intestinal expression of the proinflammatory cytokines IL-1β and IL-6 (ELISA) at 4 dpi after SBI. N.T., not treated. **C** TUNEL staining of the colons of C57BL/6 J mice treated with DMSO (control) or PJ34 (1 mg/kg) at 4 dpi. **D** Quantification of TUNEL signals in the intestines after SBI at 0 and 4 dpi. The apoptotic index was calculated by counting the TUNEL signals in 50 randomly selected crypts. The values are presented as the means ± SDs (*n* = 5 in each group). **E** PJ34 (3 μM) decreased PAR levels and reduced both cell apoptosis and the phosphorylation of STING. **F**, **G** The colocalization of STING and PAR in BMDMs after IR was repressed with 1 μg/ml PJ34. **H** STING-Alexa 488 and PAR-Alexa 647 staining of C57BL/6 J mouse intestines treated with DMSO (control) or PJ34 (1 mg/kg) at 4 dpi. **I** Quantification of the colocalization of STING-Alexa 488 and PAR-Alexa 647 in the intestine after SBI at 0 and 4 dpi. N. D., Not detected. The data are presented as the means ± SEMs (*n* = 5; two-way ANOVA; *, *p* < 0.05; ***, *p* < 0.001; ****, *p* < 0.0001).

An appropriate dose of PJ34 can also reduce the expression of intestinal inflammatory cytokines after SBI (Fig. 5A, B), thereby reducing intestinal inflammation after radiation (Fig. 4G).

Intestinal apoptosis was significantly increased after IR and was blocked by PJ34 (Fig. 5C, D and Figure. S4D). Ionizing radiation induced STING phosphorylation (Fig. 5E and Figure. S3G), which was markedly reduced by the PARP inhibitor (Fig. 5E). While IR promoted the cleavage of the apoptosis marker PARP1 (Fig. 2F and Fig. 5E), PJ34 blocked PARP1 cleavage (Fig. 5E).

The interaction between STING and PAR in the presence or absence of PJ34 after IR was further investigated. PJ34 significantly reduced the binding of STING and PAR after irradiation in BMDMs (Fig. 5F, G). The expression of STING and PAR in intestinal villous cells significantly increased after irradiation, and partial colocalization of STING and PAR was noticeable (Fig. 5H, I and Figure. S4E), which was inhibited by PJ34 (Fig. 5H, I).

We further investigated LGR5 expression in intestinal crypts with and without PJ34 treatment after IR. The results revealed that PJ34 maintained the survival of most LGR5^+^ cells until 3 days after IR; however, the crypts in the untreated mice lost most LGR5^+^ cells (Figure. S4F).

These results confirmed the important role of PAR-STING in the apoptosis pathway induced by ionizing radiation. These results suggest that an appropriate amount of PJ34 inhibits the overproduction of PAR and reduces the interaction between STING and PAR, thereby reducing the phosphorylation of STING and its signaling pathways, and promoting DNA damage repair and cell regeneration.

### STING deficiency or PARP inhibition blunts DNA-sensing-mediated NF-κB and IFN-I activation upon IR

**Fig. 6:**
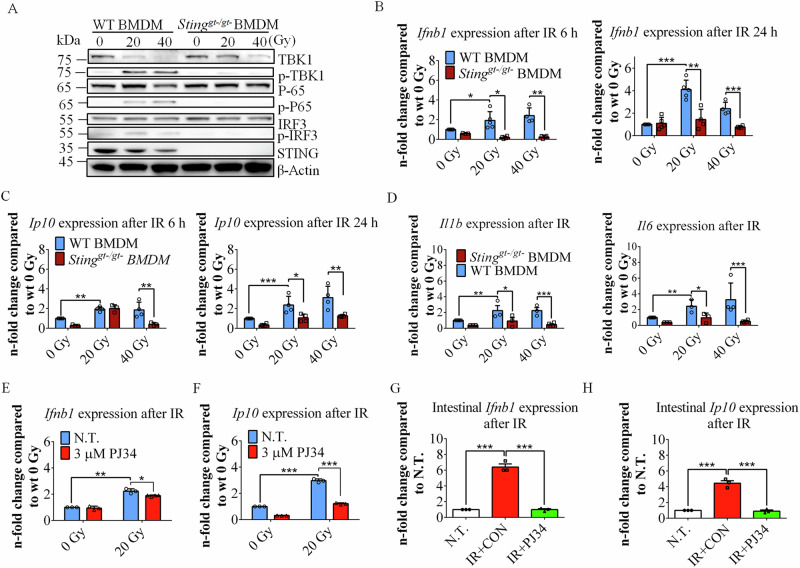
STING deficiency or PARP inhibition blunts the DNA-sensing-mediated pathway upon IR. **A** Immunoblotting analysis of TBK1, P65, and IRF3 phosphorylation in WT and *Sting*^*gt-/gt-*^ BMDMs after IR. **B**, **C** Evaluating the expression levels of *Ifnb1* and *Ip10* after IR in WT and *Sting*^*gt-/gt-*^ BMDMs via qRT‒PCR. The data are presented as the means ± SEMs (*n* = 5; two-way ANOVA; *, *p* < 0.05; **, *p* < 0.01; ***, *p* < 0.001). **D** Evaluating the expression levels of *Il1b* and *Il6* at 6 hpi in WT and *Sting*^*gt-/gt-*^ BMDMs via qRT‒PCR. **E**, **F** Expression of the cytokines *Ifnb1* and *Ip10* after IR in BMDMs treated with 3 μM PJ34. **G**, **H** PJ34 blunts the intestinal expression of *Ifnb1* and *Ip10* at 4 dpi after SBI. N.T., not treated; CON, treated with DMSO as a control; The data are presented as the means ± SEMs (unpaired Student’s t test; *, *p* < 0.05; **, *p* < 0.01; ***, *p* < 0.001).

Activated STING translocates from the ER to the Golgi apparatus, where it triggers the activation of TANK-binding kinase 1 (TBK1). TBK-1 then mediates phosphorylation-dependent activation of interferon regulatory factor 3 (IRF3) [20–22]. In addition, STING activates nuclear factor kappa B (NF-κB) through activation of the IκB kinase (IKK) complex [25]. To investigate the effects of the IR-mediated activation of the DNA sensors STING and PARP1 on the IRF3 and NF-κB pathways, we evaluated the expression levels of key genes in these pathways. Immunoblot analysis revealed that the phosphorylation of TBK1, IRF3 and P65 was abolished in *Sting*^*gt-/gt-*^ BMDMs after IR (Fig. 6A). Similar results were also observed in THP-1 cells (Figure. S3A). These results confirmed that both the IRF3 pathway and the NF-κB pathway were attenuated in STING-deficient cells.

The expression levels of *Ifnb* and the IFN-I-dependent cytokine *Ip10* were also decreased in STING-deficient cells after irradiation (Fig. 6B, C). Like those in BMDMs, *IFNb* and *IP10* expression was significantly lower in *STING*^*-/-*^ THP-1 cells than in WT THP-1 cells after IR (Figure. S5A, B). These results confirmed that STING deficiency reduced both *Ip10* and *Ifnb* expression after IR.

We further investigated the NF-κB-mediated expression of proinflammatory cytokines. The expression of the proinflammatory cytokines *Il6 and Il1b* increased after radiation, and STING deficiency significantly decreased proinflammatory cytokine expression after IR (Fig. 6D).

We additionally examined the role of PAR in mediating STING-dependent activation of IFN-I during IR. The expression of *Ip10* and *Ifnb* decreased in the presence of the PARP inhibitor PJ34 after IR (Fig. 6E, F). These results indicate that PAR produced by the DNA sensor PARP1 may be required for facilitating STING-mediated activation of IFN-I upon IR exposure. We further investigated the expression of these cytokines in the radiation-induced DNA-sensing pathway in mouse intestinal tissues. The PARP1 inhibitor significantly blunted the expression of *Ip10* and *Ifnb* in the intestine after IR (Fig. 6G, H).

Since the phosphorylation of STING also activates the NF-κB pathway and results in the production of proinflammatory cytokines, we further examined the expression of proinflammatory cytokines. Although 3 μM PJ34 significantly reduced the expression of the proinflammatory cytokines *Il6* and *Il1b* after IR, high concentrations increased the expression of these cytokines in BMDMs (Figure. S5C). PARP1 inhibition also decreased the expression of the intestinal proinflammatory cytokines IL-6 and IL-1β induced by DNA damage after IR, as shown previously (Fig. 5A, B, Figure. S5D).

These results suggest that the loss of STING function or the inhibition of PARP1 function can alleviate intestinal inflammation after IR by reducing the production of proinflammatory factors. It is possible that STING deficiency or PARP inhibition attenuates the DNA-sensing-mediated NF-κB pathway and reduces the expression of proinflammatory factors [39].

### Loss of STING function or PARP function inhibits the activation of the proapoptotic family of proteins and Bax translocation to mitochondria after irradiation

**Fig. 7:**
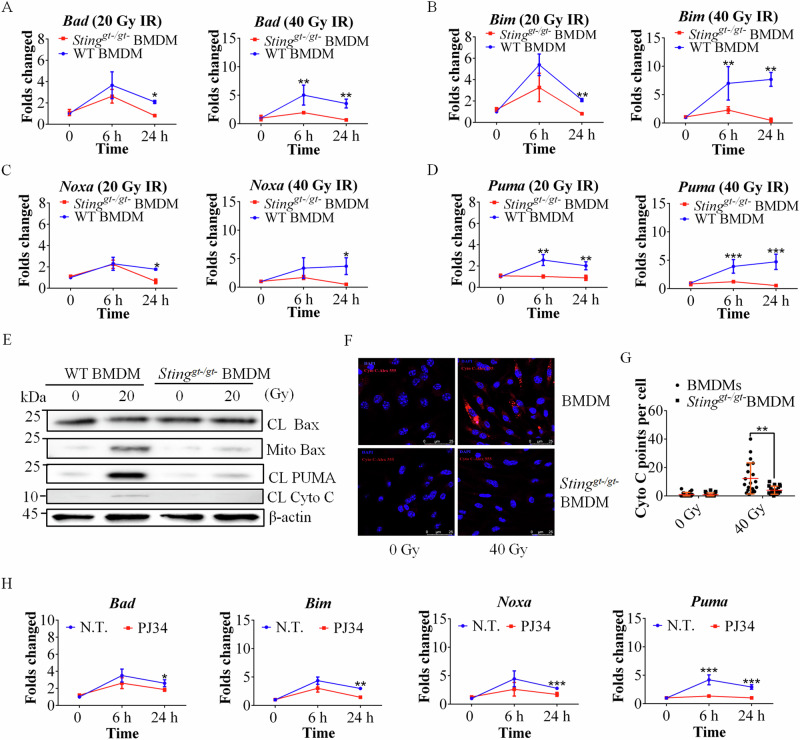
IR-mediated apoptosis increased mitochondrial Bax in a STING-dependent manner. **A‒D** Evaluating the expression of BH3 proapoptotic proteins in WT and *Sting*^*gt-/gt-*^ BMDMs after IR (20 Gy or 40 Gy) by qRT‒PCR. Fold changes and n-fold changes were compared to those of untreated WT BMDMs. **E** Immunoblotting analysis of mitochondrial Bax in WT and *Sting*^*gt-/gt-*^ BMDMs after IR, as evaluated by Western blotting. CL: cell lysate, Mito: mitochondrial protein. **F** Microscopy images of cytochrome C in WT and *Sting*^*gt-/gt-*^ BMDMs with and without IR. **G** Quantification of the level of cytochrome C via ImageJ software. **H** Quantification of BH3 proapoptotic protein expression in WT BMDMs treated with 3 μM PJ34 or vehicle at the indicated time points after IR (20 Gy) by qRT‒PCR. When the folds changed, the n-fold change was compared to that of N.T. (untreated). The data are presented as the means ± SEMs (two-way ANOVA; *, *p* < 0.05; **, *p* < 0.01; ***, *p* < 0.001).

In addition to the expression of proinflammatory cytokines and IFN-I, the expression of BH3 family proteins has been reported to be regulated by the NF-ĸB signaling pathway, and STING deficiency results in the downregulation of these proteins [24]. Therefore, we investigated the expression levels of proapoptotic genes, including *Puma*, *Bim*, *Noxa* and *Bad*, after IR. Indeed, qRT‒PCR analysis revealed that the expression of these proapoptotic genes was significantly lower in *Sting*^-/-^ BMDMs and THP-1 monocytes than in WT control cells after IR (Fig. 7A‒D, Figure. S5E‒F). Notably, the expression of the proapoptotic gene *Puma* was significantly lower in STING-deficient cells than in other proapoptotic genes (Fig. 7D, Figure. S5F). Compared with that in WT cells, PUMA expression was lower in STING-deficient cells after IR (Fig. 7E and Figure. S5G).

PUMA has been demonstrated to directly activate Bax [40, 41]. Our data confirmed that the expression of PUMA after radiation was associated with Bax expression (Figure. S5H). Furthermore, we showed that the downregulation of PUMA prevented apoptosis through the inhibition of Bax translocation to mitochondria (Fig. 7E and Figure. S5G).

To address whether the IR-mediated activation of STING promotes the translocation of Bax from the cytoplasm to the mitochondria, we fractionated BMDMs after IR. As shown in Fig. 7E, the expression level of Bax was lower in the mitochondrial fraction of *Sting*^*gt-/gt-*^ cells than in that of WT control cells (Fig. 7E). These results indicated that STING activation triggered by IR-mediated apoptosis induced BAX translocation to the mitochondrial-outer membrane (MOM). The release of mitochondrial cytochrome c (Cyto C) into the cytosol triggers programmed cell death [42]. Therefore, we evaluated the level of Cyto C after IR. Indeed, the level of Cyto C released from mitochondria after IR was significantly lower in *Sting*^*gt-/gt-*^ cells than in WT control cells (Fig. 7E–G and Figure. S5G).

Furthermore, we examined the expression levels of the proapoptotic genes *Puma*, *Bim*, *Noxa* and *Bad* after IR in BMDMs with and without the PARP inhibitor PJ34. Our data confirmed that these four proapoptotic genes play a role in IR-induced apoptosis; in particular, *Puma* expression was significantly decreased by PJ34 after IR (Fig. 7H).

These results confirmed that impaired PAR-STING pathway activity blunts the PUMA-Bax-mediated apoptotic signaling pathways after IR.

## Discussion

In this study, we revealed a novel role for STING in IR-mediated cell death in both in vitro and in vivo mouse models. Our results indicated that the loss of STING function was associated with the suppression of radiation-mediated apoptosis and was also protective against GIS characterized by prolonged survival in mice and reduced intestinal crypt damage. We found that the STING response to IR-mediated DNA damage occurs via direct binding of STING to PAR, which results in apoptosis. Phosphorylation of STING plays a key role in innate immune activation and the apoptosis pathway [22, 24, 35]. Our results also revealed that the phosphorylation of STING mediated by IR, PAR or the STING agonist diABZI promotes apoptosis.

In addition, we found that the intracellular PAR level plays an important role in the survival of cells or mice after IR. PAR is necessary for DNA repair [43], and PAR inhibition decreases UV-induced cell death [44]. Previous reports have shown that different concentrations of a PARP1 inhibitor lead to different results [37], where the administration of 3.2 mg/kg PJ34 reduced cortical injury by 33%; however, when the concentration was increased to 10 mg/kg, the degree of injury improved by only 17%. Our results also revealed a correlation between the PAR concentration and cell survival after IR. PJ34 (3 μM) decreased the IR-mediated induction of PAR. In contrast, neither low nor high concentrations (30 μM) of PJ34 could protect cells against high doses of IR. These results indicate that an appropriate amount of PAR is crucial for DNA repair and cell survival after radiation. These results also indicate that if PARP1 inhibitors are used to treat tumors, sufficient doses are required to completely inhibit the production of PAR to kill tumors.

Loss of STING function is associated with the downregulation of proapoptotic proteins [24]. The NF-κB signaling pathway, which is activated by STING [25], regulates the expression of the BH3 family of proteins and the proapoptotic BH3-only family of proteins [45]. PUMA is a direct target of NF-κB and an essential protein for DNA damage-induced apoptosis [46]. However, the underlying mechanisms of this process remain unclear [5, 47]. Our data also demonstrated that the upregulation of the proapoptotic gene PUMA was significantly reduced after IR in STING-deficient cells. Kang et al. recently showed that PUMA deficiency enhances the DNA repair process and significantly improves the survival of mice subjected to a lethal dose of IR [48]. Puma expression results in the activation of Bax and rapidly induces apoptosis in cells lacking the BH3-only proteins Bid and Bim [40, 42, 49]. In addition, an impaired Puma‒Bax axis resulted in STING-deficient cells and increased cell viability.

The effects of ionizing radiation on DNA damage occur mainly through direct ionizing energy and indirect effects, such as the generation of ROS by IR, which act on water molecules. Our data suggest that ROS production after lethal doses of radiation is not significantly affected by STING deficiency. The lethal dose irradiation may directly produce substantial DNA damage, which then directly activating the PAR-STING signaling pathway. Furthermore, the amount of IR-induced ROS may be much greater than the amount of ROS produced by other pathways after IR [50, 51], or it may be that the ROS-induced apoptotic pathway is independent of STING after lethal irradiation [52, 53]. In the future, investigating whether different radiation doses affect ROS production and different cell death pathways or patterns would be worthwhile [54].

It have been reported that PAR is located at the DNA damage foci and induces the DNA repair or cell death [11, 13]. However, how exactly PARP1 and PAR or PARylation contribute to the formation and organization of DNA repair condensates remains unclear [55]. Proteins such as ATR, ATM, DNA-PK, BRCA, RAD51, etc., play an important role in DNA damage repair process [15]. ATM and ATR trigger diverse cellular responses to DNA damage or stalled DNA replication, and activation of ATM by isorhamnetin protects mice from radiation GIS [56]. BRCA1 and BRCA2 play a role in DSBs repair by homologous recombination (HR) [57], the HR related DNA repair protein RAD51 can protect intestine against lethal dose of radiation [58]. High Mobility Group Box 1 protein (HMGB1) is a DNA chaperone that is involved in key biological processes such as DNA transcription, replication, repair, and recombination [59], STING activated by HMGB1 lead to radiation-induced liver disease [60]. Whether these proteins aggregate at radiation-induced DNA damage sites associated with PAR to initiate DNA repair or cell death would be a very interesting study.

Type I interferons (IFN-Is) are known to induce apoptosis [61]. IP10, a chemokine secreted from both IFN-I- and IFN-II-stimulated cells, is involved in the induction of apoptosis in ovarian cancer cells [62]. We have also shown that the expression levels of both *Ifnb* and *Ip10* are attenuated in STING-deficient cells. This may partly explain the lower level of IR-mediated apoptosis in STING-deficient cells. However, Takemura et al. reported that the survival rate, crypt death rate, and gastrointestinal syndrome (GIS) rate were not significantly different between mice lacking the IFN-I receptor (IFNAR) and WT mice after IR [63]. With or without IR, more cell death was observed upon treatment with the STING agonist diABZI. Compared with IR alone, diABZI activated the STING pathway and induced high expression of *Ifnb* and *Ip10* but not the inhibitor C178 (Figure. S5I). This may explain why diABZI led to exacerbated cell death after IR, as *Ifnb* and *Ip10* may help cause more cell death. The expression of proinflammatory cytokines is decreased by STING deficiency or PARP1 functional inhibition, alleviating intestinal inflammation after IR and thereby improving the GIS.

Mitochondrial DNA released into the cytoplasm upon viral infection activates the IFN-I signaling pathway in a STING-dependent manner. The noncanonical NF-ĸB pathway inhibits radiation-induced IFN-I activation mediated by STING [64]. In addition, blocking the noncanonical NF-ĸB pathway promotes antitumor immunity after radiotherapy [65]. Dunphy et al. reported that etoposide-induced DNA damage triggers noncanonical STING activation in a cGAS-independent manner and promotes NF-ĸB signaling [66]. We found that cGAS knockout did not affect PAR-induced STING phosphorylation compared with that in primitive cells (Figure. S5J), indicating that PAR-induced STING can be independent of cGAS. In the future, further identification of the circumstances under which they play a role is necessary.

DNA damage can activate STING to enhance T-cell antitumor immunotherapy, and direct activation of STING through STING agonists can promote tumor cell apoptosis and antigen surface display, thereby improving the efficacy of immunotherapy [26, 27]. The inhibition of STING activity can reduce STING-mediated inflammatory cytokines [67]. PARP-1 has been reported to play an important roles in various inflammatory diseases [68]. The discovery of the PAR-STING signaling mechanism will provides a theoretical basis for the treatment of tumors or inflammatory diseases through the interaction of these two targets.

Overall, our study revealed a novel pathway through which STING regulates IR-mediated cell death by directly binding to PAR. Therapeutically targeted STING or PARP1 may have therapeutic applications for controlling the GIS upon exposure to high levels of ionizing radiation or radiation therapy against cancers.

## Materials and methods

### Animals

The experiments were performed in accordance with the Institutional Animal Care and Use Committee guidelines from the University of California–Los Angeles and NIH guidelines. Eight- to twelve-week-old wild-type C57BL/6 J and *Sting*^*gt-/gt-*^ animals purchased from Jackson Laboratory and bred at the UCLA animal facility were used for this study. The animals were maintained and bred under specific pathogen-free conditions in the UCLA-DLAM mouse facility, and experiments were performed according to our approved protocol guidelines [69].

### Mouse irradiation

To irradiate the mice, we followed the protocol described by Micewicz et al. [69] with slight modifications. Briefly, mice anesthetized with ketamine/xylazine were placed in pie-shaped chambers with Cerrobend (1 cm) shielding of the entire body except the abdomen, 50 cm from the radiation source with a dose rate of 237 cGy/min and 320 kVp X-rays (Gulmay, Surrey, UK) for SBI. The irradiation dose was measured and calculated using an ionization chamber. A 320 Biological Irradiator (Madison, CT) was used for irradiation. Animal health was monitored and body weight was assessed daily after irradiation. Euthanasia was carried out by exposure to carbon dioxide, and the euthanasia criteria for irradiated mice included weight loss (up to 20%), dyspnea, decreased mobility, difficulty in obtaining food or water, hunching back, prolonged sleepiness, bloody or excessive diarrhea for more than 2 days, inability to remain upright, and decreased physical condition (BCSs from 3 to 2). All experimental mice were euthanized after IR for no more than 40 days.

### Primary cells and cell lines

BMDMs were harvested from the femurs and tibias of 6–8-week-old C57B/L6 mice (Jackson Labs) and differentiated in 6-well plates (1.5 × 10^6^ cells per well) with Dulbecco’s modified Eagle’s medium (DMEM; Thermo Fisher) supplemented with 1% penicillin/streptomycin, 10% FBS, and 2% MCSF or 10 ng/ml macrophage colony-stimulating factor (M-CSF) for 7 days at 37 °C in a 5% CO_2_ humidified atmosphere [18]. The media was replaced every other day, and on day 7, the differentiated cells were subjected to IR. Wild-type and *Sting*^*-/-*^ J2 virus-transformed macrophages (*Sting*^*-/-*^ J2 BMDMs) were cultured in DMEM supplemented with 10% FBS and 1% penicillin/streptomycin at 37 °C in a 5% CO_2_ humidified atmosphere. *Sting*^*-/-*^ MEFs reconstituted with STING-GFP were kindly provided by Dr. Nan Yan (University of Texas Southwestern Medical Center) [70] and cultured in DMEM supplemented with 10% FBS and 1% penicillin/streptomycin at 37 °C in a 5% CO_2_ humidified atmosphere. THP-1 and *STING*^*-/-*^ THP-1 cells, a kind gift from Dr. Robert Modlin at UCLA [71], were cultured in RPMI 1640 (Thermo Fisher) supplemented with 10 mM HEPES (pH 7.8), 10% FBS, and 1% penicillin/streptomycin and were maintained in a 5% CO_2_ incubator at 37 °C at an approximate density of 1 × 10^6^/ml. All cells are free from mycoplasma contamination.

### Reagents and antibodies

Anti-STING (D2P2F) antibody (13647), anti-BAX (2DE11) antibody (5023), anti-PARP (46D11) antibody (9532), anti-cleaved PARP (Asp214)(D64E10) antibody (5625), anti-PAR(E6F6A) antibody (83732), phospho-STING (Ser 365/Ser 366) antibody, anti-Puma (D30C10) antibody (12450), anti-Myc-tag (9B11) antibody (2276), anti-TBK1 (E813G) antibody (38066), phospho-TBK1 (Ser 172) (D52C2) antibody (5483), anti-NF-ĸB p65 (D14E12) antibody (8242), phospho-NF-ĸB p65 (Ser 536) (93H1) antibody (3033), anti-IRF3 (D6I4C) antibody (11904), and phospho-IRF3 (Ser396) antibody (29047) antibodies were purchased from Cell Signaling Technology (Denver, MA). Lipofectamine 2000 was obtained from Thermo Fisher Scientific. Poly (ADP-ribose) was purchased from Enzo Biochem, Inc. (NY, USA). cGAMP, diABZI and C178 were purchased from MedChemExpress Co. (NJ, USA). Phosphatase inhibitor cocktail I was purchased from Abcam, Inc. (Cambridge, UK). A 2,7-dichlorodihydrofluorescein (H2DCF-DA) probe was purchased from Sigma‒Aldrich (MO, USA). Fluorophore-conjugated secondary antibodies were purchased from Fisher Scientific (MA, USA). PJ34 was purchased from Selleckchem (TX, USA).

### Cell death and LDH release assay

To evaluate IR-mediated cell death after the cells were subjected to IR, the cells were stained with trypan blue, and the percentage of cell death was quantified with an automated cell counter (Thermo Fisher Scientific Inc., MA, USA). In addition, the supernatants from the IR-treated cells were harvested, and the lactate dehydrogenase (LDH) level [72] was measured via a CytoTox 96® Nonradioactive Cytotoxicity Assay Kit (Promega, G1780). The cytotoxicity was calculated as follows: Percent cytotoxicity = 100 × Experimental LDH Release (OD_490_)/Maximum LDH Release (OD_490_).

### Immunofluorescence and transmission electron microscopy

The cells were cultured on chamber slides (Thermo Fisher 177445) and harvested at the time points indicated in each experiment after irradiation. The cells were fixed for 15 min in 4% formaldehyde, blocked in blocking buffer (1 × PBS, 10% anti-goat serum, 0.3% Triton X-100) for 45 min, and incubated with primary antibodies (1:100–1:1000) in antibody solution buffer (1 × PBS, 0.1% Triton X-100) at 4 °C overnight. The cells were then washed with 1 × PBS 3 times and stained with fluorophore-conjugated secondary antibodies (1:200) at room temperature for 1 hour. The slides were washed again 3 times with 1 × PBS, stained with 4,6-diamidino-2-phenylindole (DAPI, dihydrochloride) to visualize the nuclei, and mounted on a fluoromount [73]. The confocal images were acquired via a Leica TCS SP8 and a ZEISS Elyra - 7 confocal microscope.

The irradiated cells were blocked in 2.5% glutaraldehyde and morphologically observed via transmission electron microscopy (TEM) with a Hitachi HT-7800.

### Immunoassays

Immunoblot assays were performed by collecting cells in NP40 buffer (50 mM Tris-Cl, pH 7.4; 150 mM NaCl; 1 mM EDTA; and 1% NP40) containing complete protease inhibitors (Roche). The protein content of precleared cell lysates was quantified with a BCA assay (Thermo Scientific), and equal amounts of protein were loaded and subjected to SDS‒PAGE, followed by blotting to PVDF membranes (Millipore). Immunoprecipitation was performed as described previously [73]. Briefly, precleared cell lysates were incubated with appropriate antibodies at 4 °C, followed by the addition of protein A agarose beads (Roche) at 4 °C. The captured proteins were eluted with 2× Laemmli sample buffer (Bio-Rad) and subjected to SDS‒PAGE, followed by Western blotting.

### Protein purification and binding of the sting and PAR

Vector pET28b constructs carrying the human *STING1* gene were transformed into strain BL21 (DE3). The transformants were diluted by 1:1000 and cultured in 500 mL of LB medium containing 50 μg/ml kanamycin at 37 °C to an absorbance at 600 nm of 0.4 and then induced with 1 mM IPTG for a 4-h incubation [74]. The cells were collected in lysis buffer (25 mM sodium phosphate (pH 8.0), 250 mM NaCl) and disrupted by an ultrasonic disruptor. Lysate in lysis buffer with 20 mM imidazole was loaded onto the Ni-NTA column, and then the tagged proteins were eluted with the same buffer containing different concentrations (20 mM, 50 mM, 100 mM, 250 mM) of imidazole. The eluted proteins were detected by SDS‒PAGE and dialyzed against 25 mM sodium phosphate (pH 7.4) and 250 mM NaCl. The protein was further purified through a Superdex 200 Increase chromatography column with 25 mm HEPES, pH 7.5, 150 mM NaCl, and 0.06% digitonin as the running buffer. All the experiments were performed at 4 °C.

The purified protein (0.2 μg) was incubated with 100 nM, 1 μM or 10 μM PAR at 37 °C for 3 h, and 10 μM PAR with phosphate buffer was used as the control.

The gel shift assay was conducted by nondenaturing polyacrylamide gel electrophoresis as described previously and with modifications [75] and was assayed by nondenaturing PAGE with 5% stacking gel (pH 6.8) and 8% (acrylamide) resolving gel (pH 8.8). A constant voltage of 120 V was applied for 2 h after the indicator entered the separating gel, after which the proteins were blotted onto the PVDF membranes (Millipore) with Towbin buffer (pH 8.3, 25 mM Tris, 192 mM glycine, and 20% methanol). Immunoblotting was performed with antibodies against STING and PAR. The primary antibodies were diluted to 1:1000 according to the product instructions of Cell Signaling Technology. Reference samples of the binding complex product were applied to the gel.

### Histopathology and scoring of surviving crypts

The intestines and colons of the IR-treated and control mice were dissected, fixed in 4% formalin, and embedded lengthwise in paraffin (Blue RiBbon; Surgipath Medical Industries). Blocks were sectioned to the level of the lumen and then stained with hematoxylin and eosin (H&E) or TUNEL for quantification of apoptotic crypt cells. IR-mediated damage to crypt cells was evaluated from an average of at least three cross sections per mouse [76]. The villus length of the small intestine was measured from the villus-crypt border to the tip of the villus.

### Quantitative real-time PCR (qRT‒PCR)

Cellular and intestinal tissue total RNA was extracted by using TRIzol reagent (Life Technologies) according to the manufacturer’s instructions and subjected to cDNA synthesis via the iScript™ cDNA Synthesis Kit (Bio-Rad). qRT‒PCR analysis was performed by using iTaq™ universal SYBR® Green supermix (Bio-Rad) and the CFX96 Touch Real-Time PCR Detection System (Bio-Rad). The relative mRNA expression levels of the target genes were normalized to those of the internal control ribosomal protein genes Rpl32, β-actin or GAPDH as described previously [77, 78]. The sequences of the oligonucleotides used in this study are listed in Supplemental Table 1.

### Enzyme-linked immunosorbent assay

The intestine samples were cut into small pieces and homogenized, and the levels of IL-6 and IL-1β were measured and quantified via enzyme-linked immunosorbent assay (ELISA). IL-6 and IL-1β produced in the intestine with and without treatment were detected with a Quantikine® ELISA Kit (R&D Systems, USA).

Two independent experiments with biological replicates were conducted. All protocols were carried out according to the manufacturer’s instructions.

### Quantification of apoptosis by flow cytometry

The cells in the plates were washed twice with EDTA-PBS followed by trypsinization (0.25% trypsin-EDTA), and the detached cells were harvested by centrifugation at 300 × g for 5 min and washed once in cold PBS containing 0.5% BSA. The harvested cells were resuspended in 1 ml of Annexin binding buffer (Thermo Scientific) [79] protected from light, incubated at room temperature for 5 min, and then transferred to 4 °C, after which propidium iodide (50 mg/ml) was added. The cells were immediately subjected to flow cytometer analysis (BD Bioscience, UK) with 488 nm and 350 nm lasers to detect apoptosis and cell death. For the apoptosis analysis, cellular debris and cell aggregates were excluded from the analysis by the application of electronic gates.

### Proximity ligation assay

The Duolink® In Situ Red starter kit mouse/rabbit (DUO92101) was purchased from Sigma‒Aldrich (St. Louis, MO, USA). The in situ proximity ligation assay was performed according to the manufacturer’s protocol [33, 80]. An anti-STING1 (human specific) mouse monoclonal antibody (D199110) was purchased from BBI Co., Ltd. (SH, CN). The rabbit antibodies anti-PARP (46D11) antibody (9532) and anti-PAR (E6F6A) antibody (83732) were purchased from Cell Signaling Technology (Denver, MA). Since the STING primary antibody used for PLA is human-specific and because in situ immunohistochemistry requires adherent cells, the MDA-MB-231 cells [26] were precultured on sterilized glass slides and treated with 20 Gy irradiation. The confocal images were acquired via a Leica TCS SP8 confocal microscope.

### Statistical analysis

All experiments were performed in triplicate, with at least two independent biological replicates. All the statistical analyses were performed with SPSS statistics software and GraphPad Prism 8.0. The means of two groups were compared via two-tailed unpaired Student’s tests to generate two-tailed P values. When three or four groups were compared, we used two-way ANOVA.

## Supplementary information

Supplemental Data For paper entitled “STING interacts directly with PAR promotes apoptosis upon acute ionizing radiation-mediated DNA damage”

Original Data

## Data Availability

All data that support the findings of this study are available from the corresponding authors on reasonable request.
