# Supplementary material for: STING directly interacts with PAR to promote apoptosis upon acute ionizing radiation-mediated DNA damage
Source: Cell Death Differ. 2025 Feb 12;32(6):1167–79. doi: 10.1038/s41418-025-01457-z (PMC12163073; doi:10.1038/s41418-025-01457-z)
Supplement: Supplementary file 1 — Supplemental Data For paper entitled “STING interacts directly with PAR promotes apoptosis upon acute ionizing radiation-mediated DNA damage” [file 41418_2025_1457_MOESM1_ESM.doc]

Supplemental Data

**For paper entitled “STING interacts directly with PAR promotes apoptosis upon acute ionizing radiation-mediated DNA damage”**

Yirong Sun^1, 2†*^, Saba R Aliyari^2†^, Kislay Parvatiyar^2,3^, Lulan Wang^2^, Anjie Zhen^2^, Wei Sun^1^, Xiaobo Han^1^, Adele Zhang^2^, Ethan Kato^2^, Helen Shi^2^, Elena De Schutter^2^, William H McBride^4^, Samuel W French^5^, Genhong Cheng^2*^

1. CAS Key Laboratory of Regenerative Biology, Guangdong Provincial Key Laboratory of Stem Cell and Regenerative Medicine, Guangzhou institutes of Biomedicine and Health, Chinese Academy of Sciences, Guangzhou 510530 China

2. Department of Microbiology, Immunology and Molecular Genetics, University of California- Los Angeles, Los Angeles CA 90095 United States of America

3. Department of Microbiology & Immunology, Tulane University School of Medicine, New Orleans, LA, United States of America

4. Department of Radiation Oncology, University of California-Los Angeles, Los Angeles, CA 90095 United States of America

5. Department of Pathology and Laboratory Medicine, David Geffen School of Medicine, University of California-Los Angeles, Los Angeles CA 90095 United States of America

Supplemental Data consisted of 5 Supplemental Figures and 1 Supplemental Table.

**Supplemental Figures**

**Supplemental Figure 1**

**
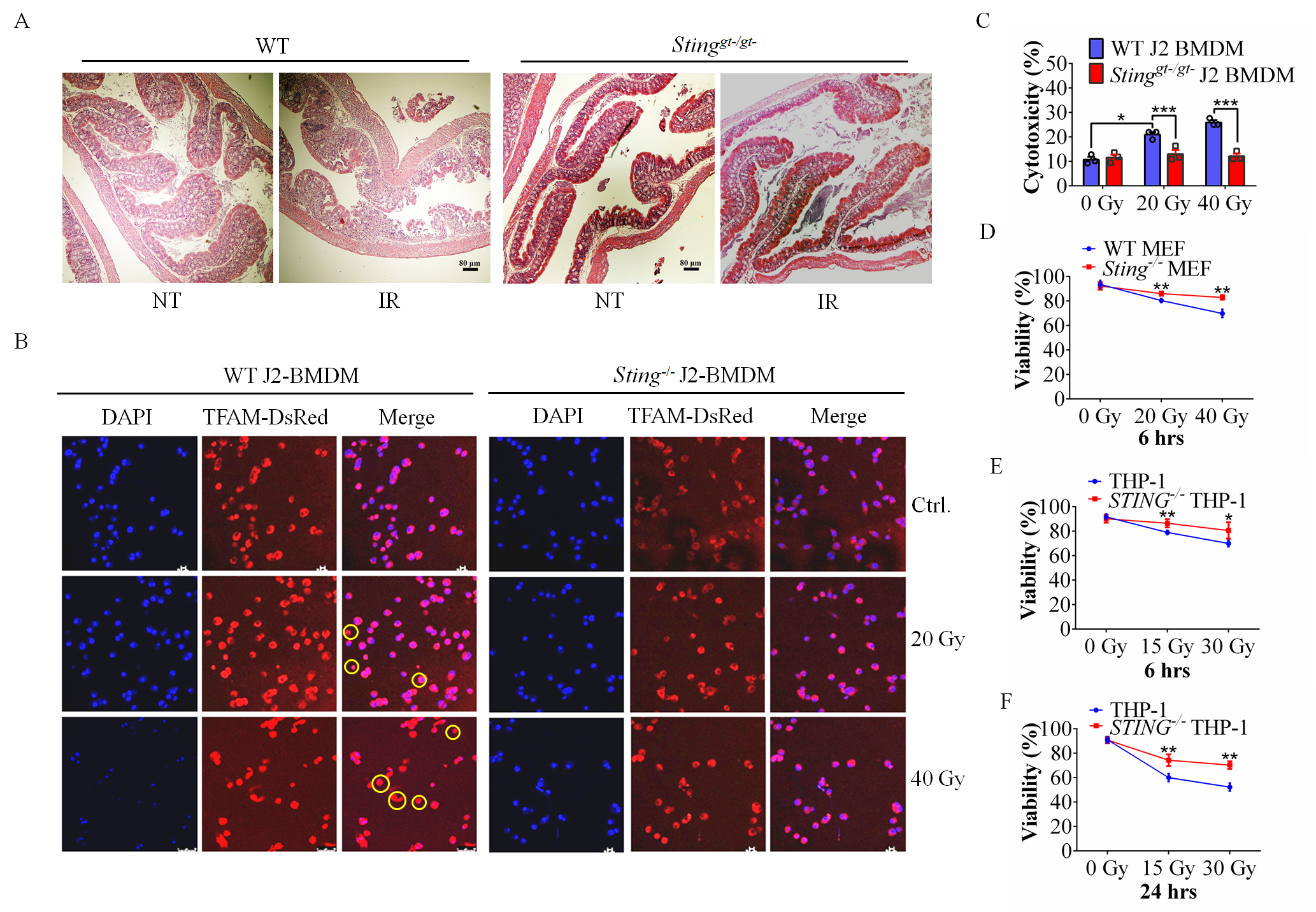
**

**Supplementary Fig. 1.** **Loss of STING function enhances resistance to IR.** **A.** H&E staining of WT C57BL/6J and *Sting^gt-/gt-^* C57BL/6J colons with (IR) and without (NT) abdominal radiation at 4 dpi (4 days post irradiation). **B.** J2 BMDMs and *Sting^-/-^* J2 BMDMs were stained with DAPI and TFAM-dsRed at 6 hpi. **C.** J2 BMDM cells viability after IR measured by LDH assay at 6 hpi. **D.** WT MEFs and *Sting*^-/-^ MEFs viability after exposure to 20 and 40 Gy rads at 6 hpi. **E-F.** Survival rate of WT and *STING*^-/-^ THP-1 cells after exposure to 15 and 30 Gy rads at 6 and 24 hpi. The data are presented as the means ± SEMs, *, p<0.05, **, p<0.01, ***, p<0.001.

**Supplemental Figure 2**

**
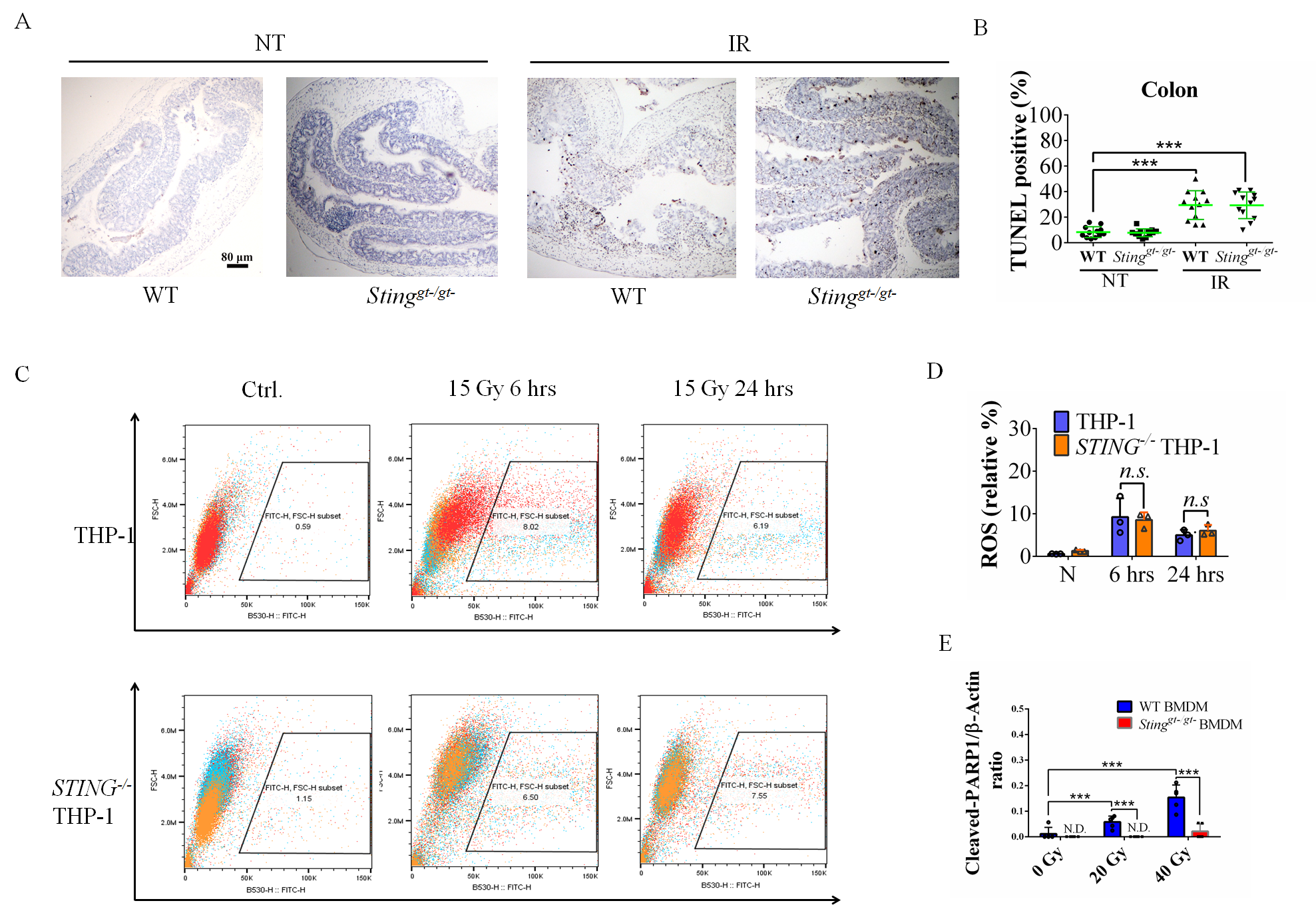
Supplementary Fig. 2**. **STING is essential for activation of IR-mediated apoptosis.** **A.** TUNEL staining of colons of WT and *Sting^gt-/gt-^* mice at 0 and 4-dpi. **B.** Quantification of TUNEL positive cells in colons after SBI at 0 and 4 dpi. **C-D.** Measuring the ROS level in WT and *STING*^-/-^ THP-1 cells after exposure to indicated dose of IR by H2DCF-DA probe. **E**. Analysis of cleaved PARP1 after IR by the gray ratio of WB. N. D., Not detected. The data are presented as the means ± SEMs (n=5; two-way ANOVA; ***, p<0.001). *n.s.*, not significant.

**Supplemental Figure 3**


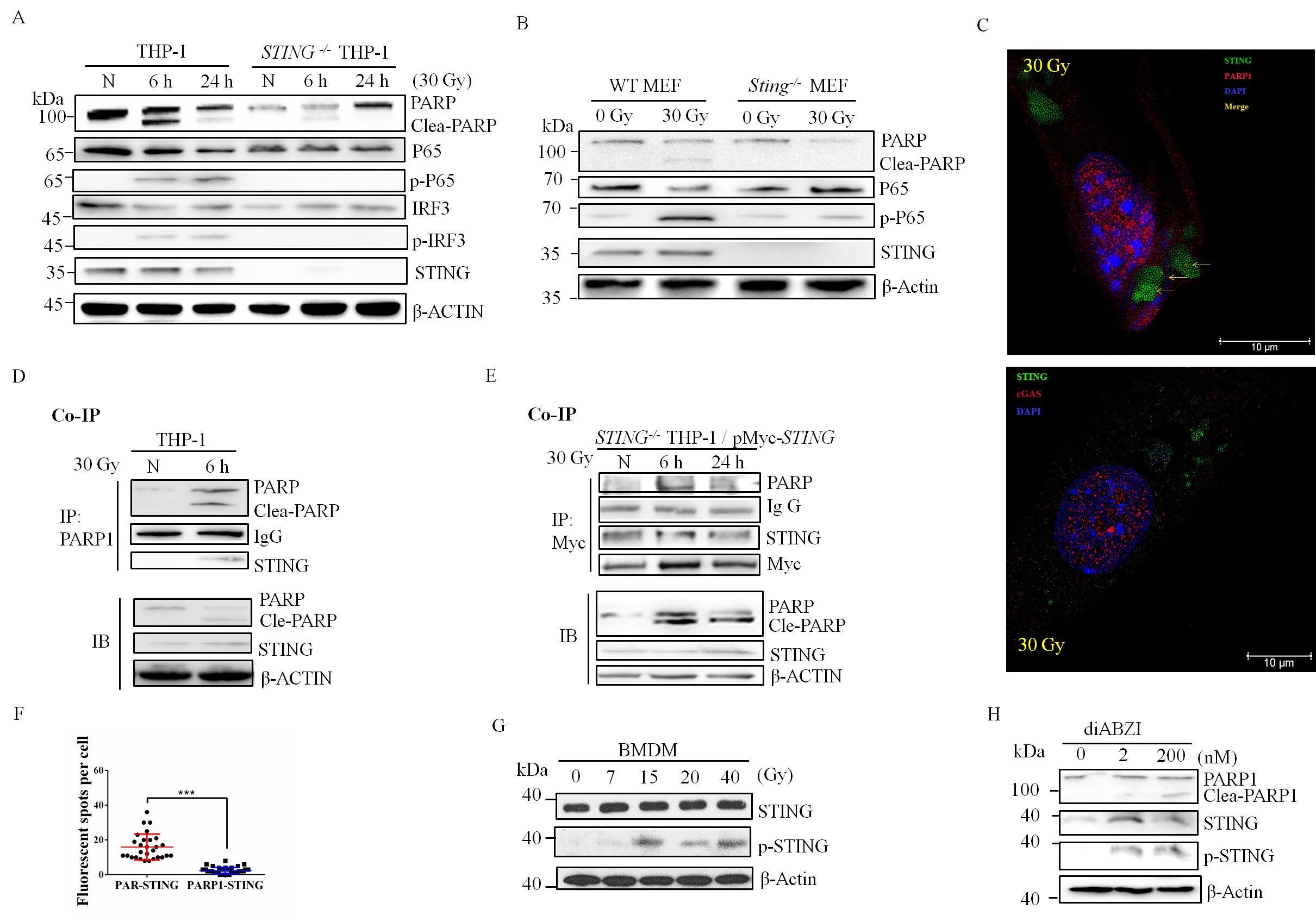


**Supplementary Fig. 3**. **STING activated after IR, association with PARP1 and PAR.** **A.** Western blot showing PARP1 cleavage and phosphorylation of P65, and IRF3 after IR in WT and *STING*^-/ -^ THP-1 cells. **B.** Evaluation of PARP1 cleavage and phosphorylation of P65 in WT and *Sting*^-/ -^ MEFs by Western blotting. **C.** Confocal images of the interactions between PARP1-Alex647 and STING-GFP or cGAS-Alex647 and STING-GFP after 30 Gy IR via a Leica TCS SP8 microscope. **D**. Association of STING with PARP1 in THP-1 cells. Cell lysates were immunoprecipitated with anti-PARP1 beads followed by immunoblotting with the indicated antibodies. **E.** Association of STING-MYC with PARP1 in THP-1 cells. Cell lysates were immunoprecipitated with anti-MYC beads followed by immunoblotting with the indicated antibodies. **F**. Relative quantification of PLA signals (discrete red fluorescent dots) of STING interacting with PAR or PARP1 in each cell after IR. Data are presented as mean ±SEM from n = 3 experiments (*** p <0.001). **G.** Immunoblotting detection of STING phosphorylation in WT BMDMs after exposure to the indicated dose of IR. **H.** The STING agonist, diABZI, induced STING phosphorylation and PARP1 cleavage at 6 hpi.

**Supplemental Figure 4**

**
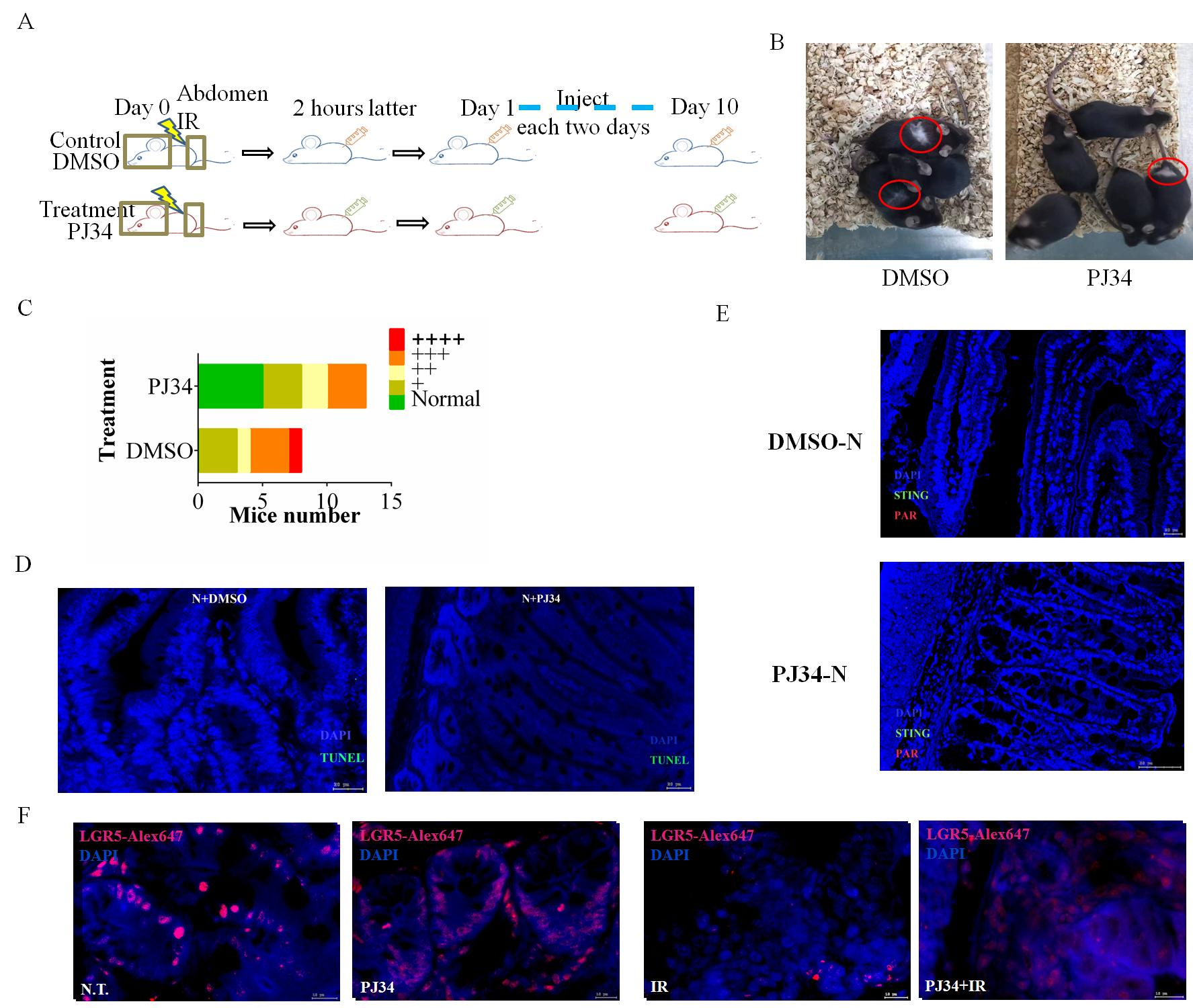
**

**Supplementary Fig. 4. Protective effect of the PARP1 inhibitor against IR *In vivo*. A.** PJ34-treated mice after 16.2 Gy SBI. Irradiated mice were injected with 1mg/kg PJ34 two hours after radiation, and for two consecutive days, followed by injection every two days until the 10th day. **B-C.** Comparison of the hair loss and abnormal behavior of mice treated with vehicle or 1 mg/kg PJ34 after receiving 16.2 Gy irradiation. “+”, Degree of depilation. **D.** TUNEL staining of intestines of C57BL/6J mice (NO IR) with PJ34 compared to the vehicle (DMSO). **E.** STING-alex 488 and PAR-alex 647 stainiing of C57BL/6J mice (NO IR) with DMSO (Control) or PJ34. **F.** LGR5 expression in crypt of intestine with and without PJ34 treatment after IR. N.T., not treatment.

**Supplemental Figure 5**

**
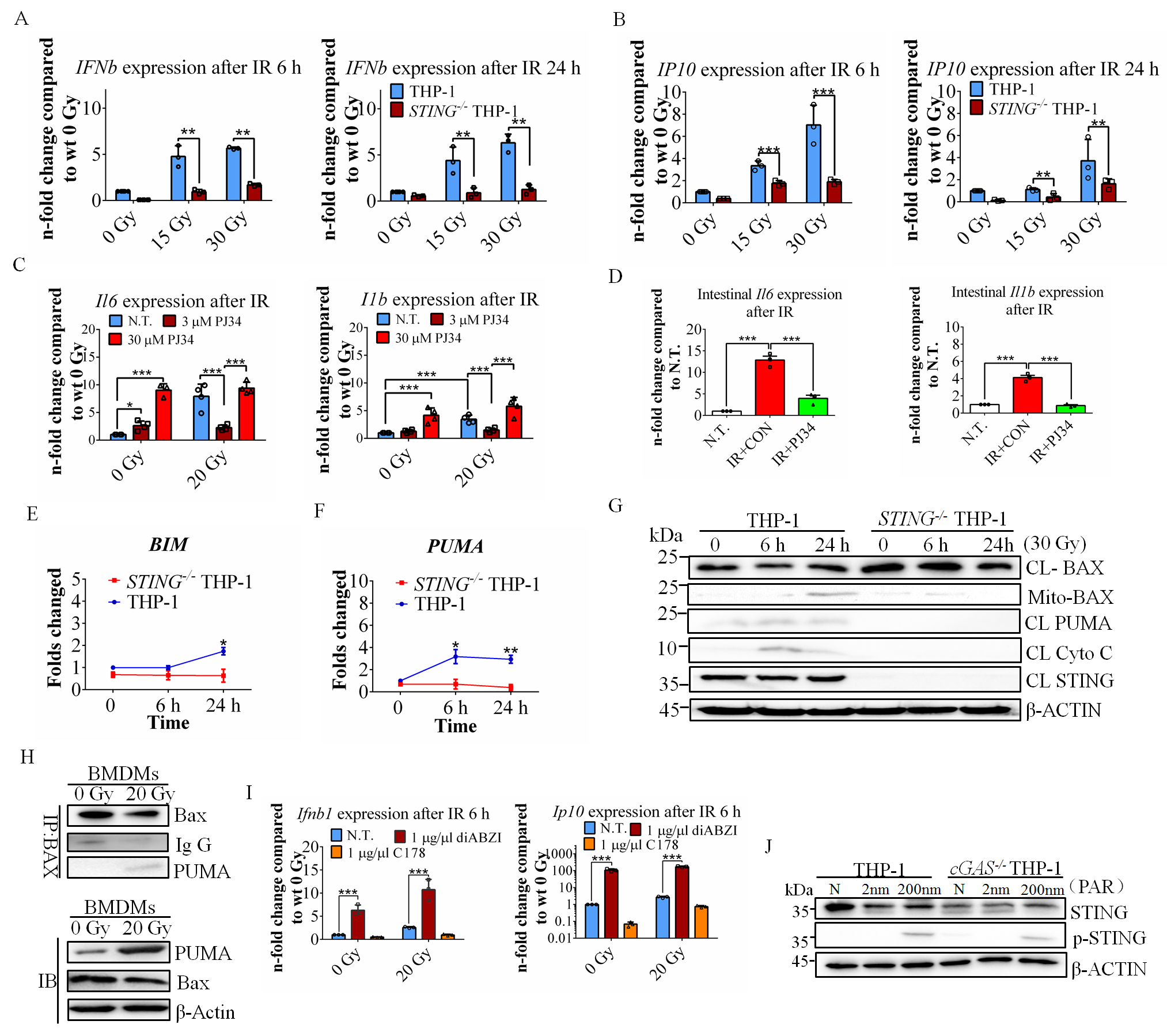
Supplementary Fig. 5**. **STING deficiency is associated with attenuation of both IRF3 and NF-κB pathways.** **A-B.** Quantification of *IFNb* and *IP10* transcripts in WT and *STING*^-/-^ THP-1 after IR by qRT-PCR. **C**. The expression of cytokines *Il1b* and *Il6* after IR in BMDMs with 3 μM and 30 μM of PJ34. **D.** PJ34 blunts the intestinal expression of the proinflammatory cytokines *Il1b* and *Il6* at 4 dpi after SBI. CON, treated with DMSO as a control. **E-F**. The expression of *BIM* and *PUMA* in WT and *Sting^-/-^* THP-1 at 6 hpi and 24 hpi measured by qPCR. **G.** Measuring the expression of mitochondrial BAX and PUMA in WT and *Sting*^-/-^ THP-1 after IR. CL: cell lysate; Mito: mitochondrial protein. **H.** Interaction of Bax with PUMA in BMDM cells. Cell lysates were immunoprecipitated with anti-Bax beads followed by immunoblotting with the indicated antibodies. **I.** The expression of cytokines *Ifnb1* and *Ip10* in BMDMs with 1 μg/ml diABZI and 1 μg/ml C178 after irradiation. **J.** THP1-Dual™ cells and THP1-Dual™ *cGAS* cells were transfected with the indicated concentration of PAR by lipofectamine for 3 hours. (THP1-Dual™ cells and THP1-Dual™ *cGAS* cells were purchased from InvivoGen). N.T., not treated; *, p<0.05, **, p<0.01, ***, p<0.001, ****, p<0.0001.

**Supplemental Table 1**
