# Supplementary material for: STING directly interacts with PAR to promote apoptosis upon acute ionizing radiation-mediated DNA damage
Source: Cell Death Differ. 2025 Feb 12;32(6):1167–79. doi: 10.1038/s41418-025-01457-z (PMC12163073; doi:10.1038/s41418-025-01457-z)

Figure 2F

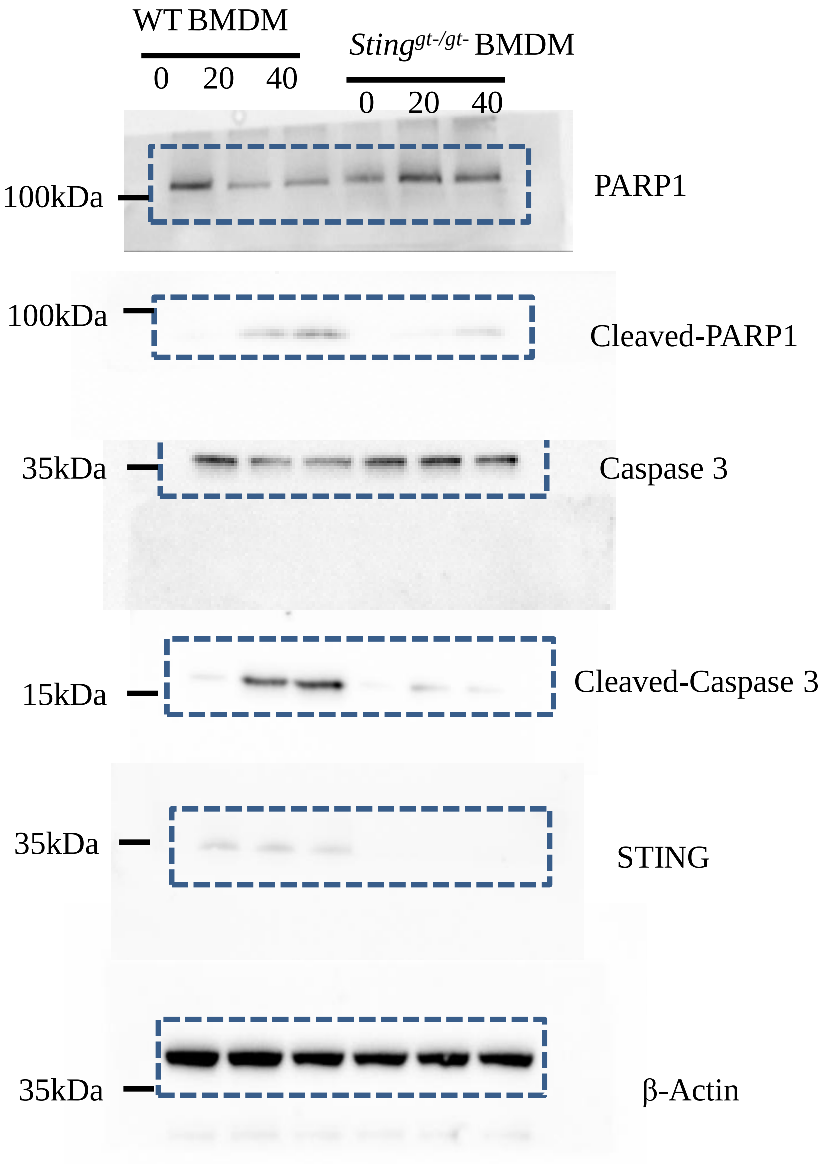

WT BMDM
0 20 40
Sting$^{gt-/gt-}$ BMDM
0 20 40
100kDa
PARP1
100kDa
Cleaved-PARP1
35kDa
Caspase 3
15kDa
Cleaved-Caspase 3
35kDa
STING
35kDa
β-Actin

Figure 3C

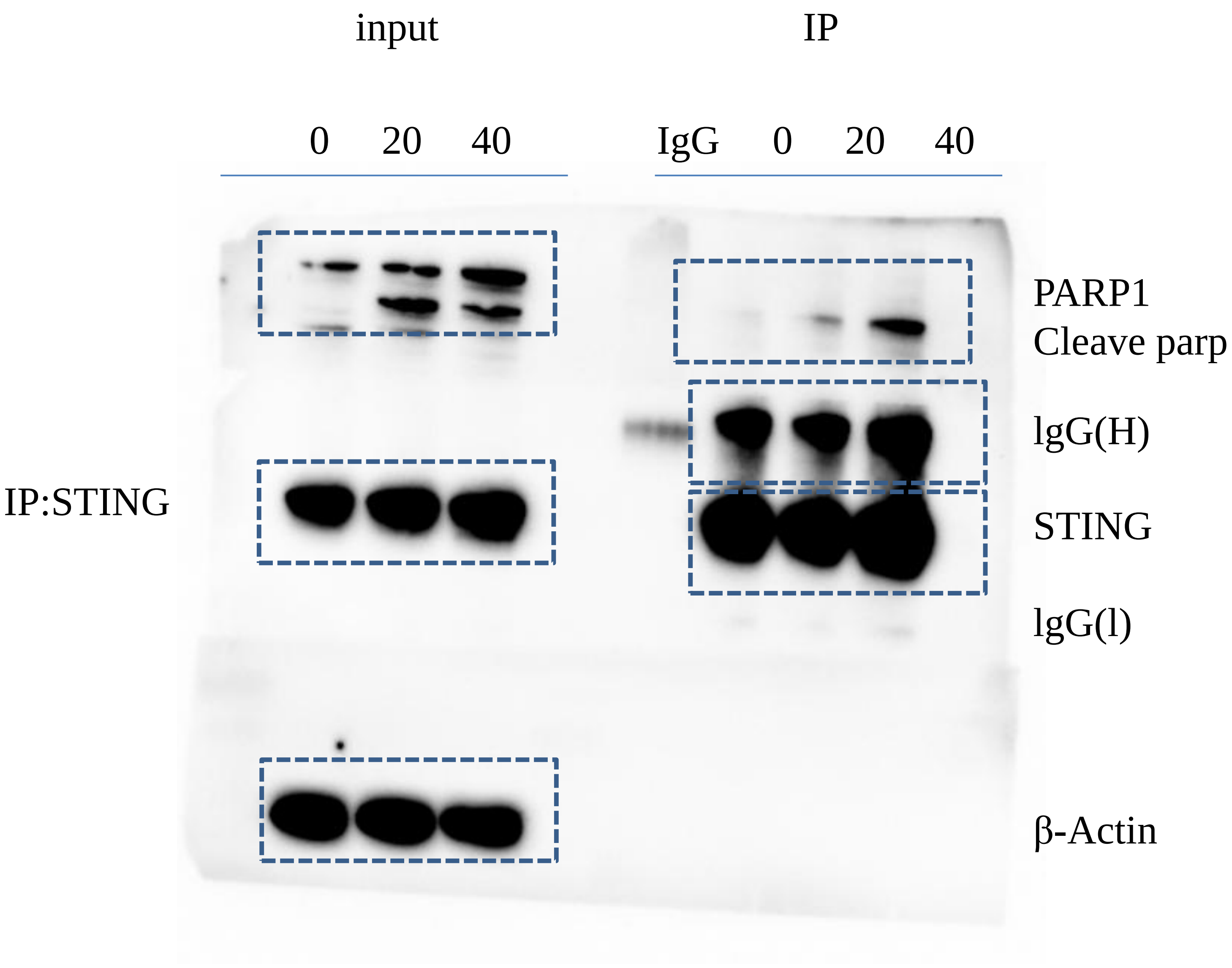

input
IP
0
20
40
IgG
0
20
40
IP:STING
PARP1
Cleave parp
lgG(H)
STING
lgG(l)
β-Actin

Figure 3D

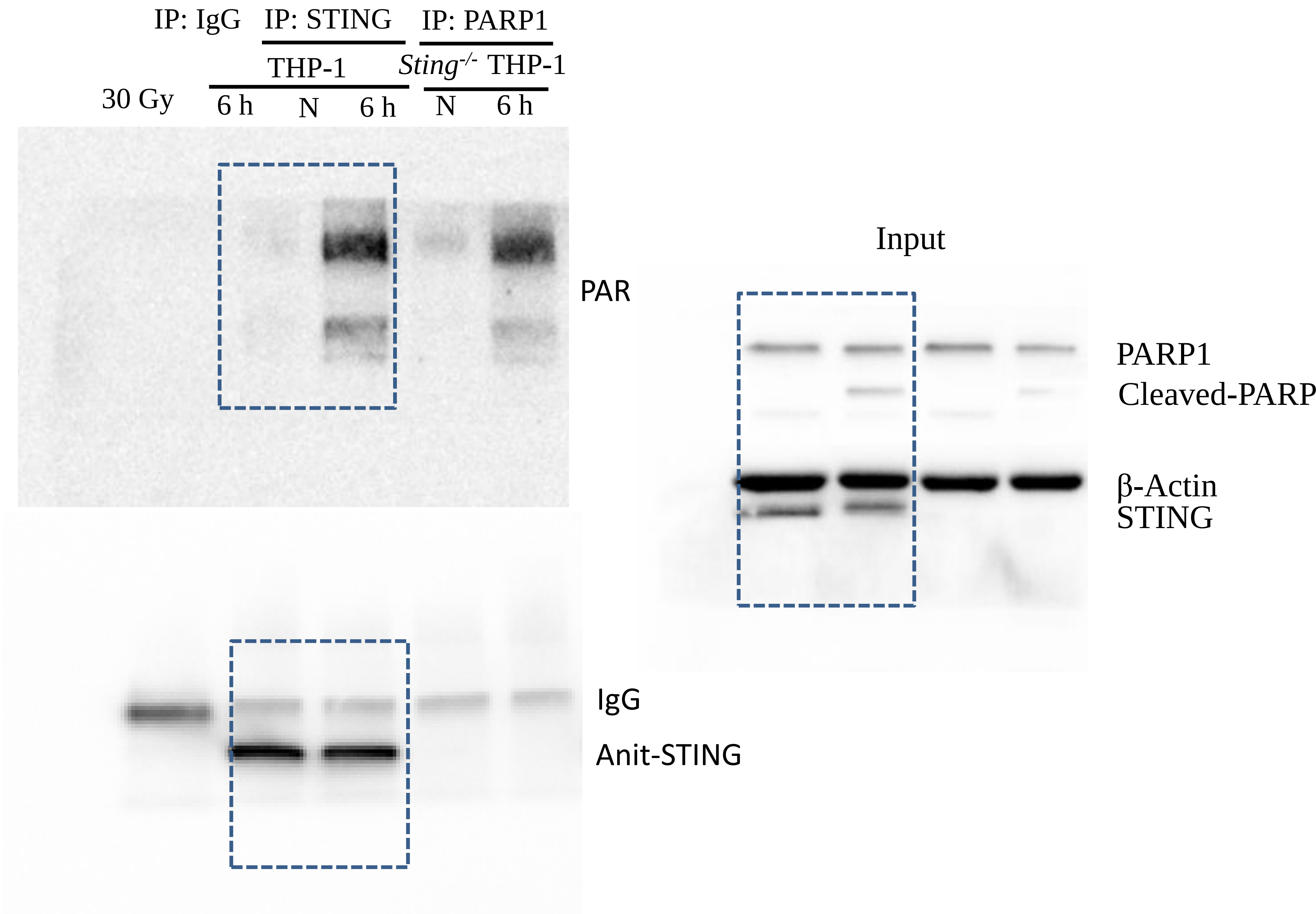

IP: IgG
IP: STING
IP: PARP1
THP-1
Sting-/- THP-1
30 Gy
6 h
N
6 h
N
6 h
PAR
IgG
Anit-STING
Input
PARP1
Cleaved-PARP
β-Actin
STING

Figure 3 G and H

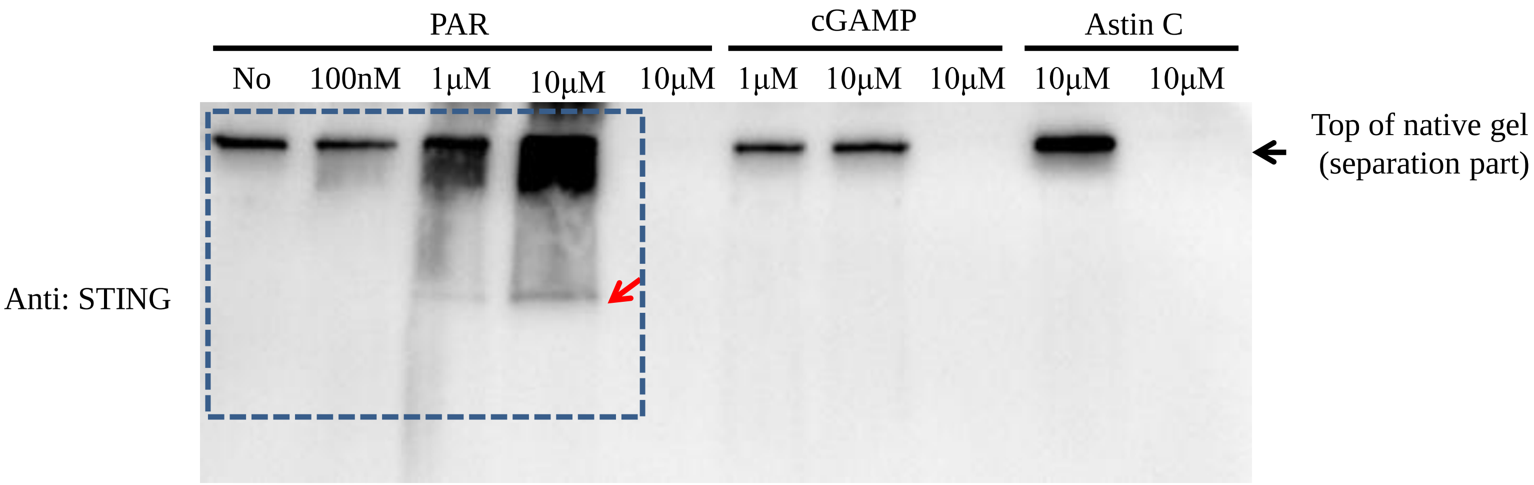

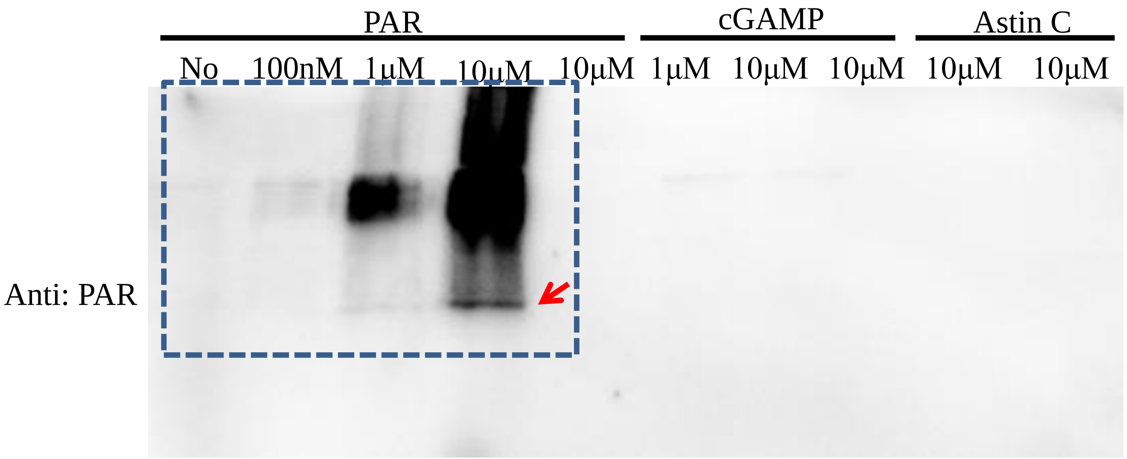

Figure 3I

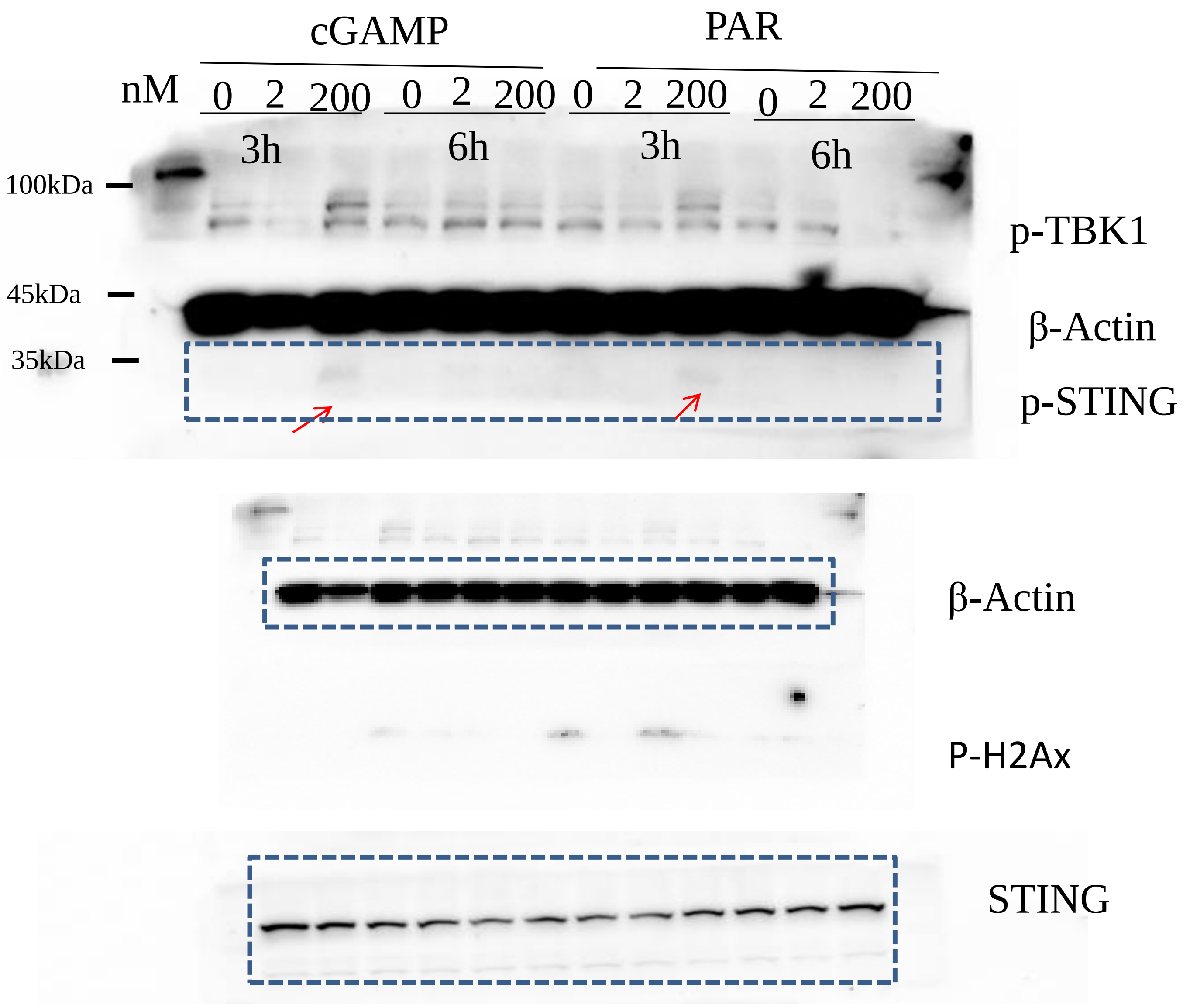

cGAMP
PAR
nM
0 2 200 0 2 200 0 2 200 0 2 200
3h
6h
3h
6h
100kDa
45kDa
35kDa
p-TBK1
β-Actin
p-STING
β-Actin
P-H2Ax
STING

Figure 4 B

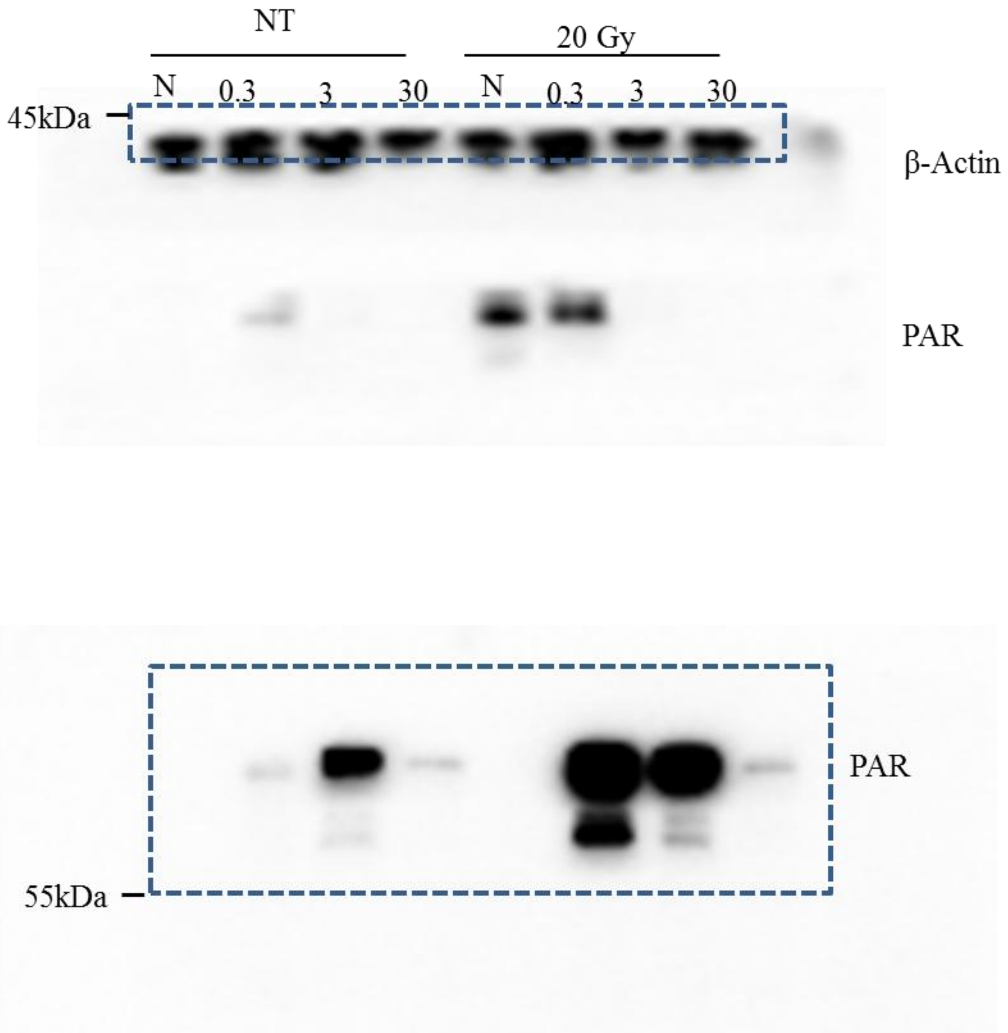

Figure 5E

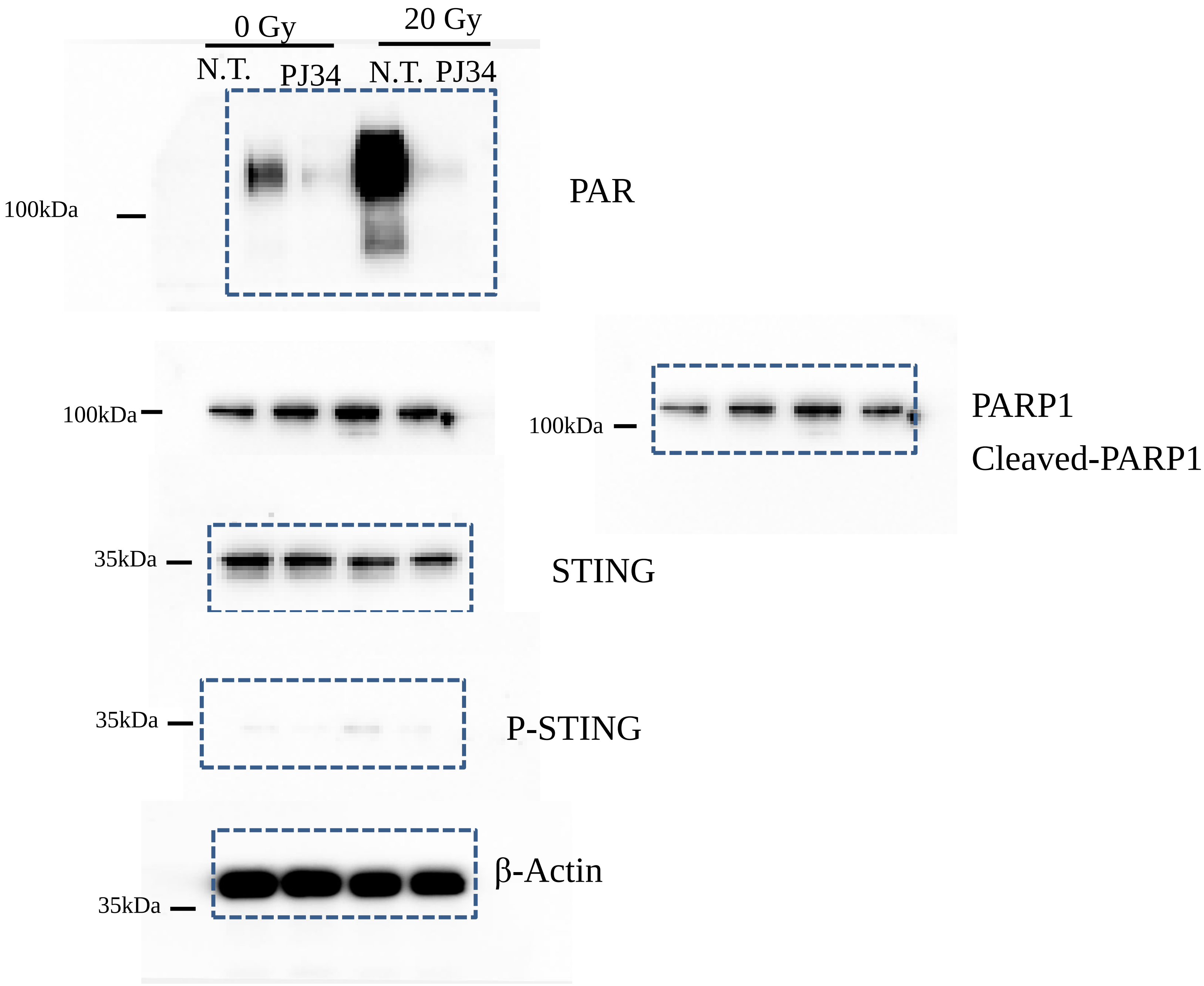

0 Gy
20 Gy
N.T.
PJ34
N.T.
PJ34
100kDa
PAR
100kDa
100kDa
PARP1
Cleaved-PARP1
35kDa
STING
35kDa
P-STING
35kDa
β-Actin

Figure 6 A

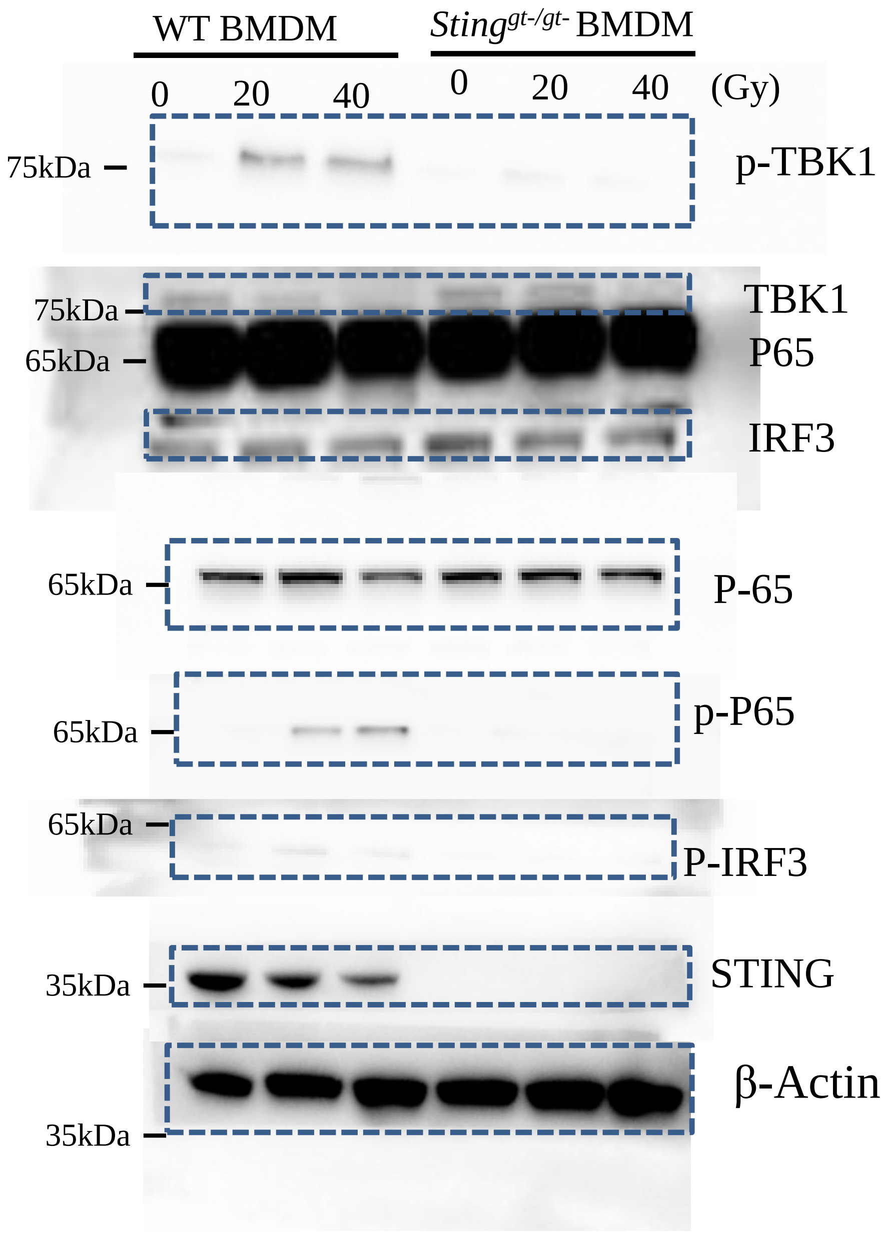

WT BMDM
*Sting*gt-/gt- BMDM
0
20
40
0
20
40
(Gy)
75kDa
p-TBK1
75kDa
65kDa
TBK1
P65
IRF3
65kDa
P-65
65kDa
p-P65
65kDa
P-IRF3
35kDa
STING
β-Actin
35kDa

Figure 7E

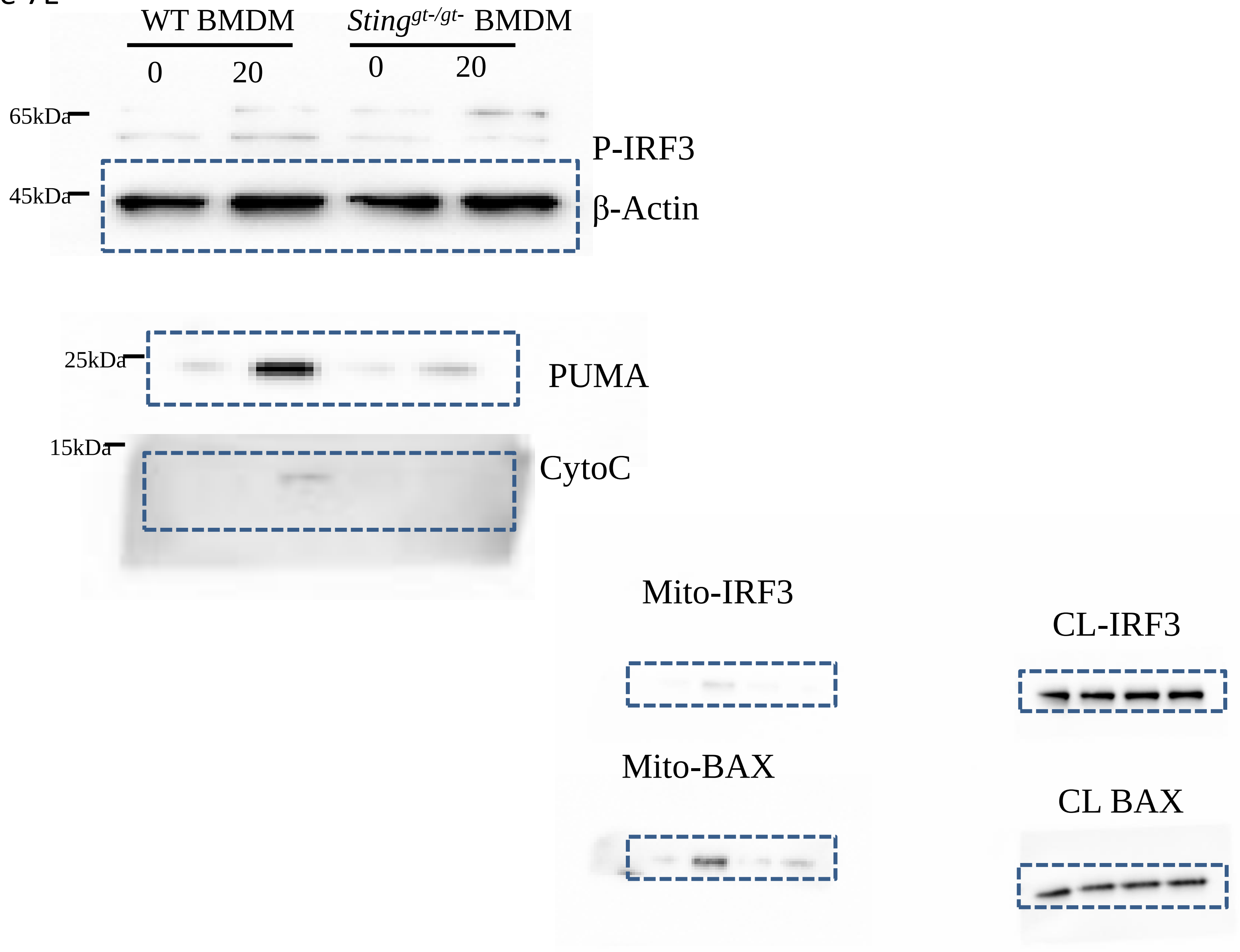
WT BMDM
Sting$^{gt-/gt-}$ BMDM
0
20
0
20
65kDa
45kDa
P-IRF3
β-Actin
25kDa
PUMA
15kDa
CytoC
Mito-IRF3
CL-IRF3
Mito-BAX
CL BAX

Figure S3 A

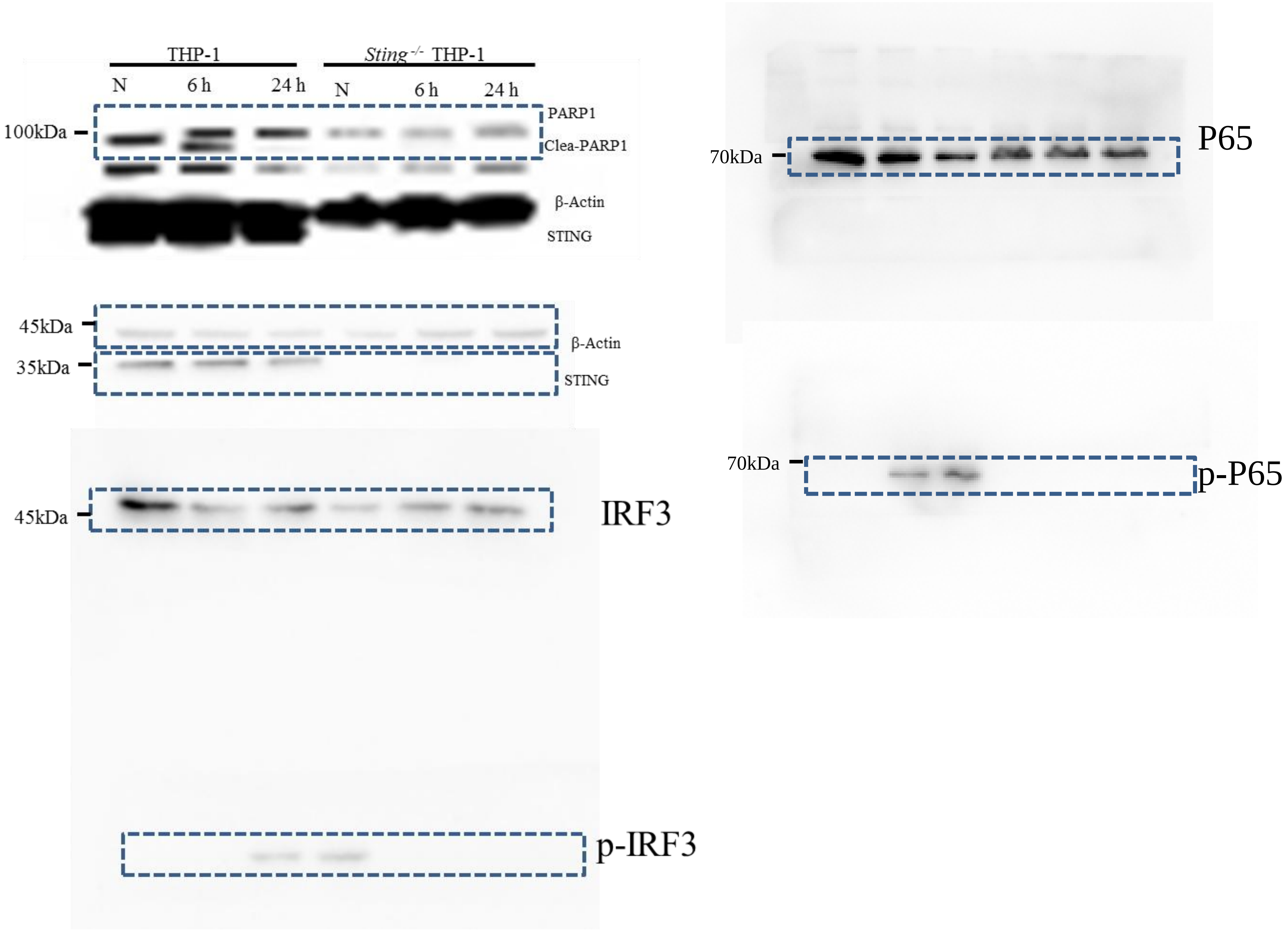
THP-1
Sting -/- THP-1
N
6 h
24 h
N
6 h
24 h
100kDa
PARP1
Clea-PARP1
β-Actin
STING
45kDa
35kDa
β-Actin
STING
45kDa
IRF3
p-IRF3
70kDa
P65
70kDa
p-P65

Figure S3 B

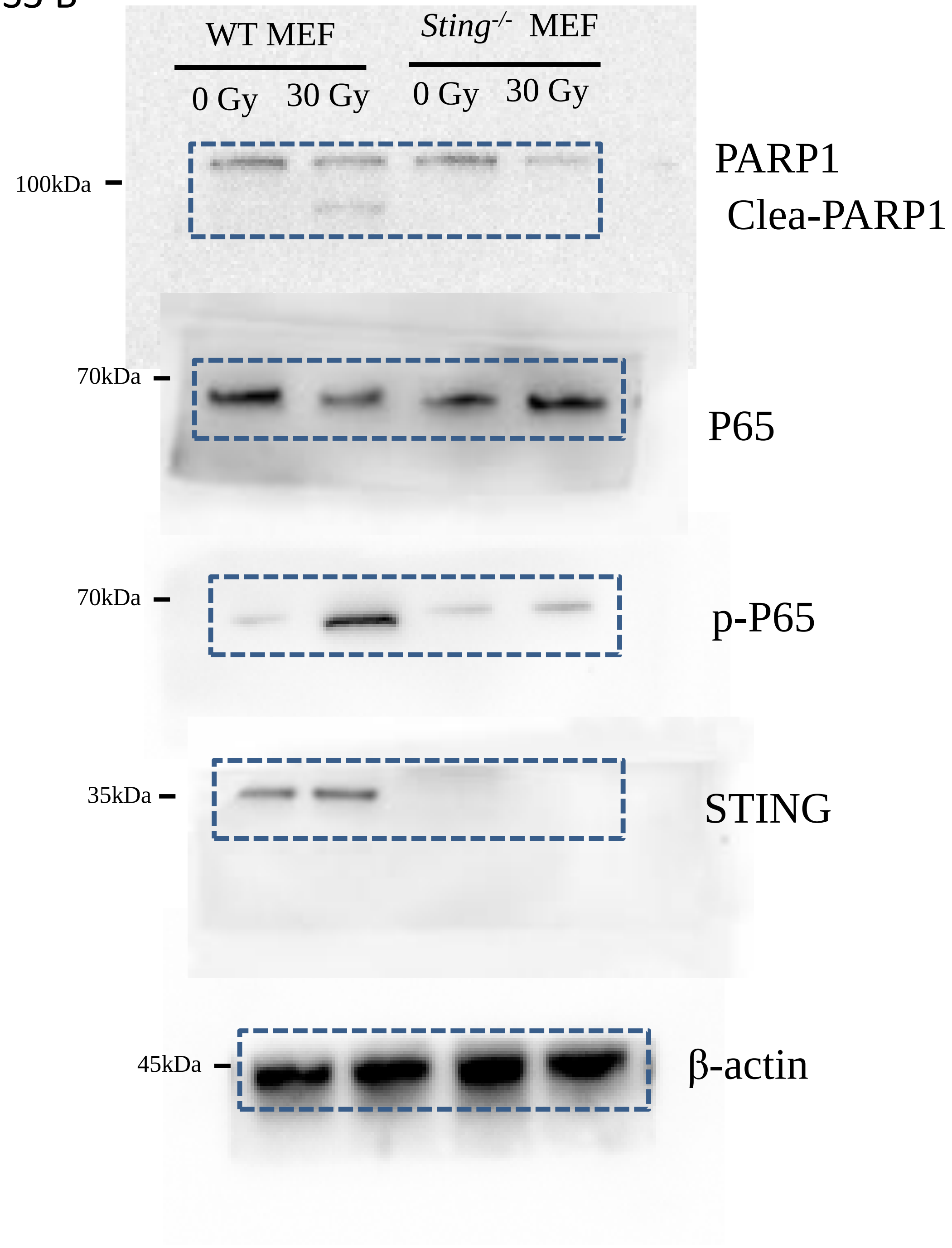

WT MEF
Sting-/- MEF
0 Gy
30 Gy
0 Gy
30 Gy
100kDa
PARP1
Clea-PARP1
70kDa
P65
70kDa
p-P65
35kDa
STING
45kDa
β-actin

Figure S3 C

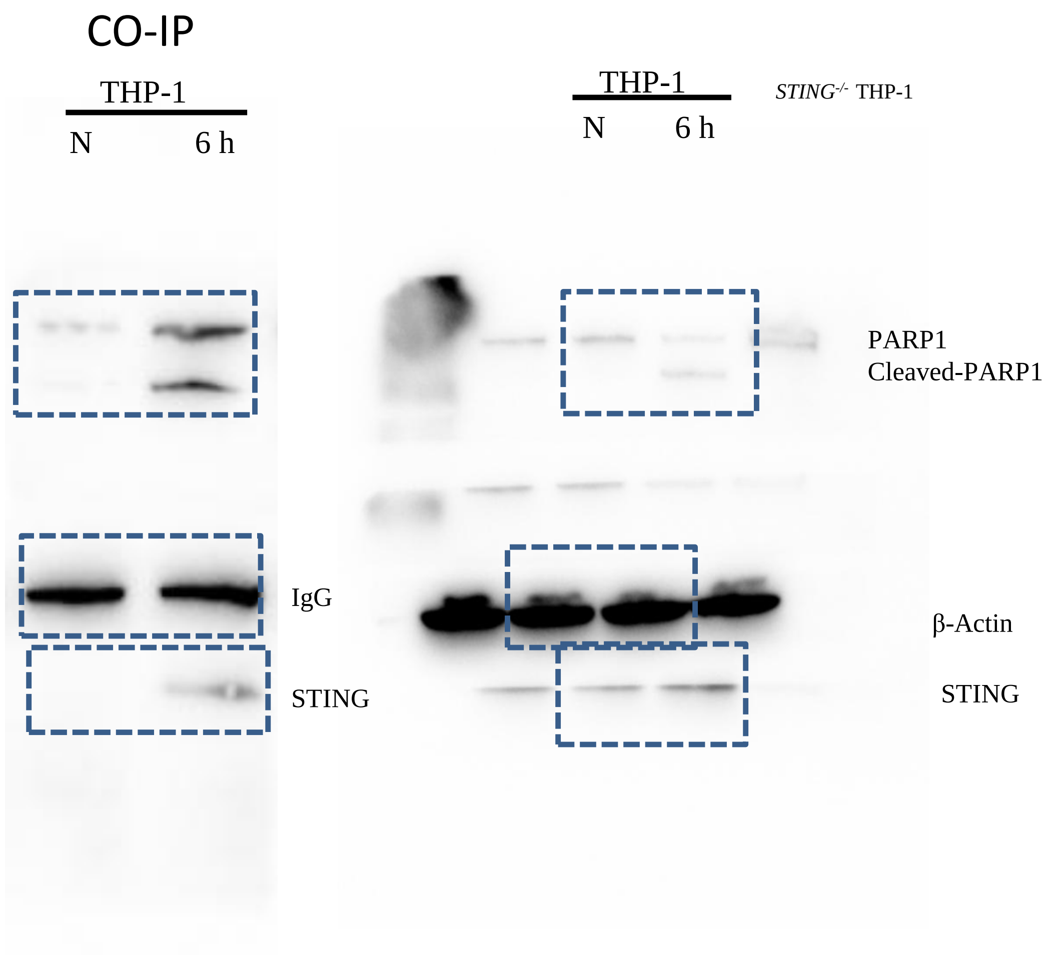
CO-IP
THP-1
N
6 h
IgG
STING
THP-1
STING-/- THP-1
N
6 h
PARP1
Cleaved-PARP1
β-Actin
STING

Figure S3 D

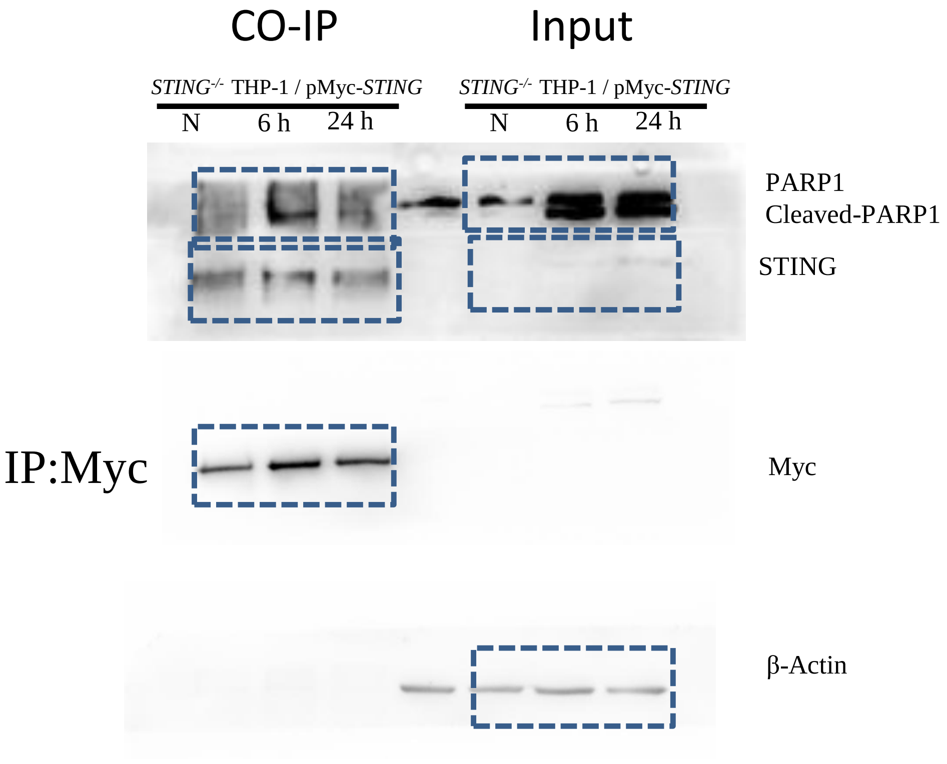

CO-IP
Input
STING-/- THP-1 / pMyc-STING
STING-/- THP-1 / pMyc-STING
N
6 h
24 h
N
6 h
24 h
PARP1
Cleaved-PARP1
STING
IP:Myc
Myc
β-Actin

Figure S3 E

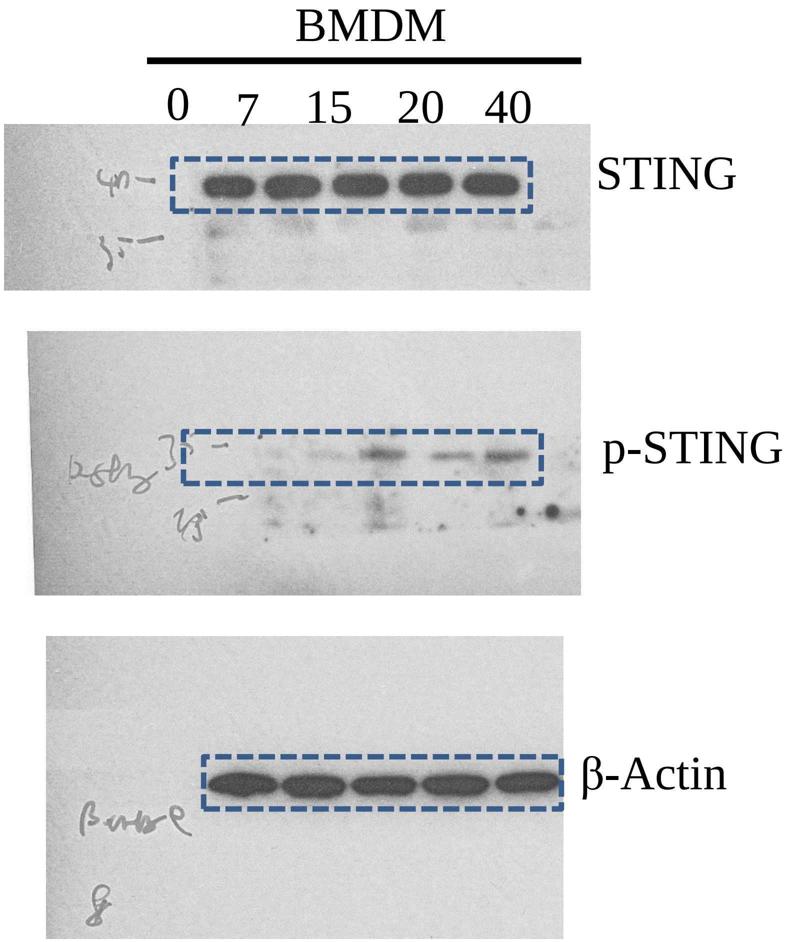

Figure S3 F

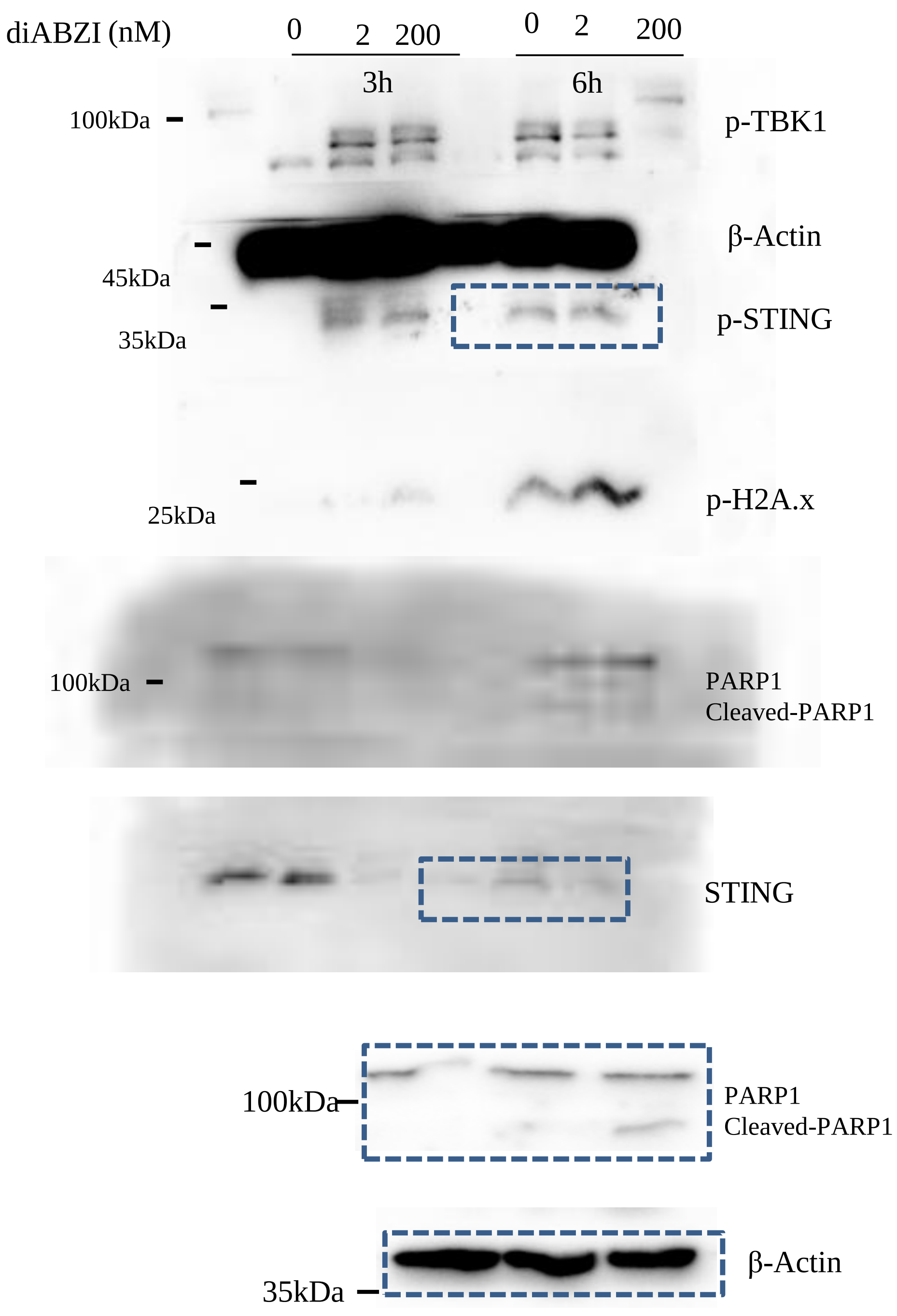
diABZI (nM)
0
2
200
0
2
200
3h
6h
100kDa
p-TBK1
β-Actin
45kDa
p-STING
35kDa
25kDa
p-H2A.x
100kDa
PARP1
Cleaved-PARP1
STING
100kDa
PARP1
Cleaved-PARP1
β-Actin
35kDa

Figure S5 H

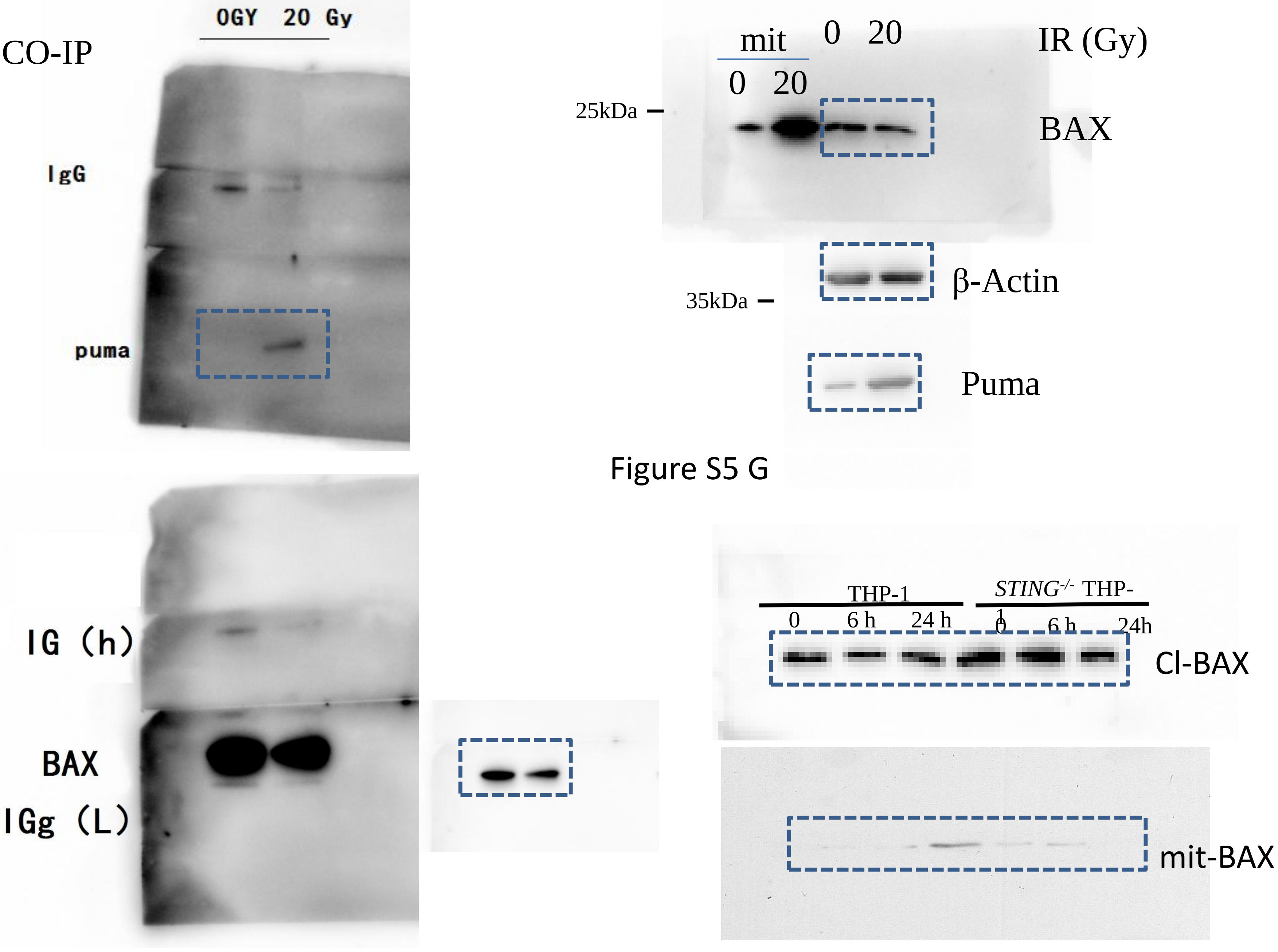

Figure S5 G

Figure S5 G

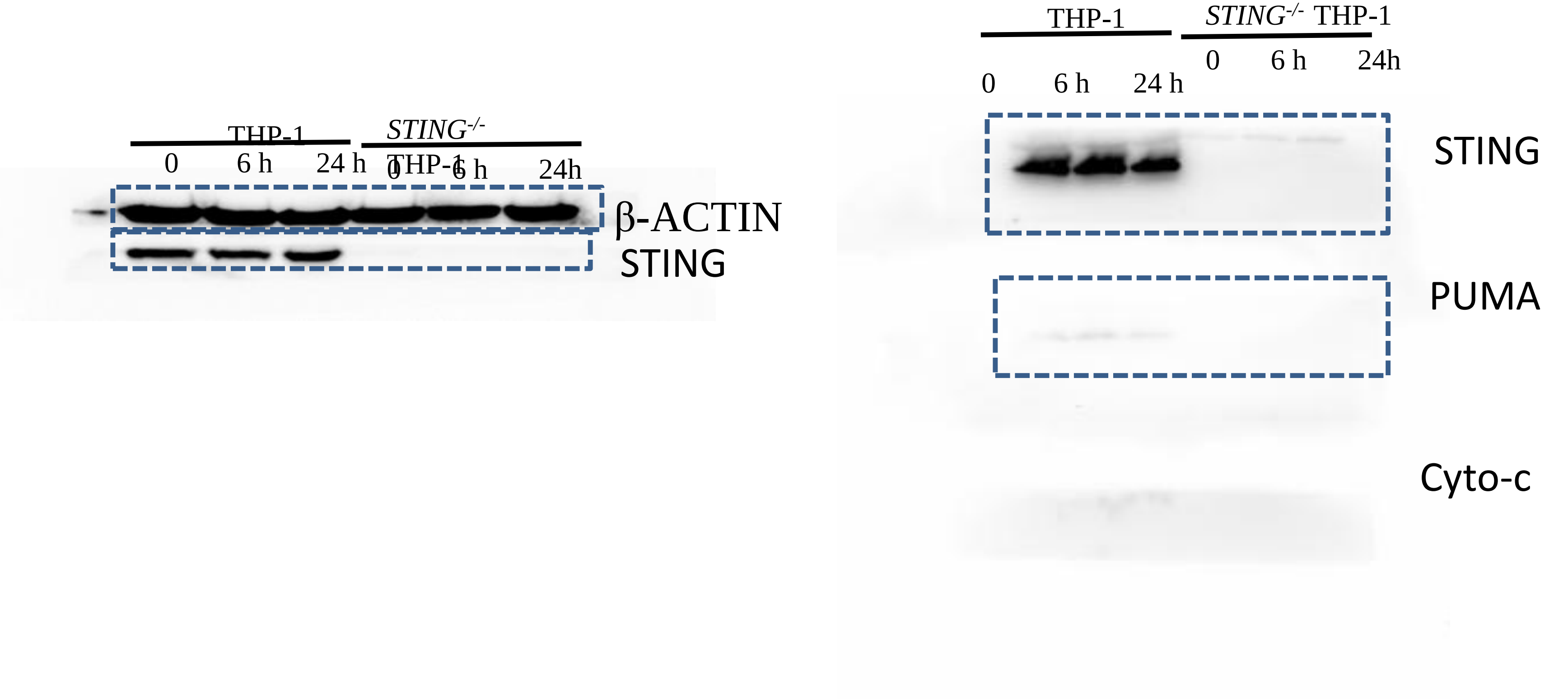

Figure S5 J

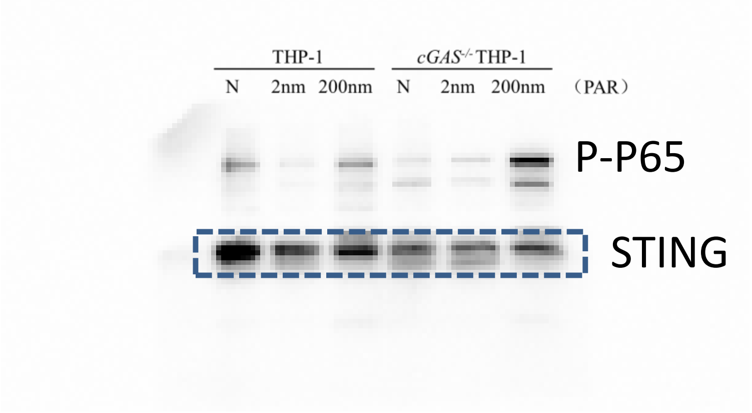

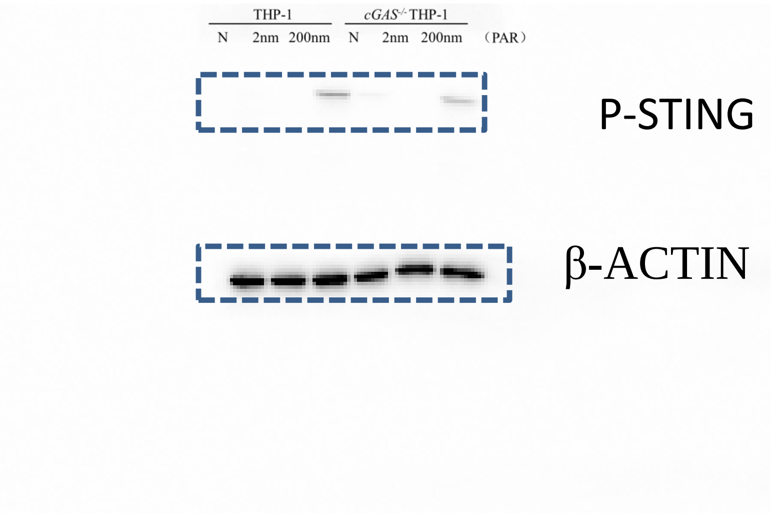

Supplement: Supplementary file 2 — Original Data [file 41418_2025_1457_MOESM2_ESM.pdf]
